# New Paratethyan dwarf baleen whales mark the origin of cetotheres

**DOI:** 10.7717/peerj.5800

**Published:** 2018-10-15

**Authors:** Pavel Gol'din

**Affiliations:** Schmalhausen Institute of Zoology, National Academy of Sciences of Ukraine, Kiev, Ukraine

**Keywords:** *Ciuciulea*, Cetotheriidae, *Otradnocetus*, Baleen whales, Miocene, Paratethys, Biogeography, Phylogeny, Taxonomy

## Abstract

**Background:**

Family Cetotheriidae *sensu stricto* and several closely related taxa comprise the Cetotherioidea and represent a lineage of Neogene baleen whales that includes the smallest edentulous baleen whales in Earth history. Most of known cetotheriids came from the Late Miocene to Quaternary, and the earliest records from the latest Middle Miocene. The Paratethys region shows a great diversity of Middle to Late Miocene cetotheriids. That includes nominative taxon of the family, *Cetotherium rathkii*, and this suggests that the earliest cetotheriids may have lived in that region.

**Materials and methods:**

Here, *Ciuciulea davidi*, a new genus and species from the Middle Miocene of southeastern Europe, is described as the chronologically earliest and earliest diverging member of Cetotheriidae. Also, a new specimen of *Otradnocetus*, a basal Cetotherioidea sensu Gol’din & Steeman, 2015 is identified from the Late Miocene deposits of Caucasus and compared with *Otradnocetus virodovi* from the Middle Miocene of the same region.

**Results and discussion:**

*Ciuciulea davidi* is a dwarf whale displaying primitive traits: posterior ends of facial bones forming a single transverse line, a narrow occipital shield, and a relatively long interparietal region. Meanwhile, it shares some cetotheriid apomorphies: posteriorly telescoped wedge-shaped facial bones and an ovoid tympanic bulla with shallow lateral and medial furrows, a short anterior lobe and a short sigmoid process. Phylogenetic analysis suggests that *Parietobalaena* and *Otradnocetus* are branches diverging before the clade Cetotheriidae + Neobalaenidae. This is confirmed by the stepwise evolution of the anatomy of the squamosal, mandible, and ear bones across these groups. The re-described juvenile specimen of *Otradnocetus* differs from *O. virodovi* in the more primitive anatomy of the mandible and the autapomorphic anatomy of the humerus. Records of the earliest cetotheriids and related taxa in the Paratethys support the idea that this could be the region where Cetotheriidae evolved before their worldwide dispersal and radiation.

## Introduction

The taxonomy and phylogeny of baleen whales of the family Cetotheriidae *sensu stricto*, along with that of other Neogene whales, was revised during the recent years (Bouetel & De Muizon, 2006; Steeman, 2007, 2010; Whitmore & Barnes, 2008; Bisconti, Lambert & Bosselaers, 2013; Fordyce & Marx, 2013; El Adli, Deméré & Boessenecker, 2014; Gol’din, Startsev & Krakhmalnaya, 2014; Kimura & Hasegawa, 2010; Marx & Fordyce, 2015; Gol’din & Steeman, 2015; Gol’din & Startsev, 2017; Marx, Lambert & De Muizon, 2017). Since 2007, seven nominal new genera and thirteen new species have been described (summarized in: Gol’din & Startsev, 2017; Marx, Lambert & De Muizon, 2017), turning this family into the best studied clade of Neogene baleen whales. The time of emergence of the family Cetotheriidae was predicted by phylogenetic analyses as the earliest Miocene (Steeman et al., 2009; Marx & Fordyce, 2015). Meanwhile, most of known cetothere records came from the Late Miocene to Quaternary (Boessenecker, 2013; Gol’din & Steeman, 2015; Gol’din & Startsev, 2017). The earliest formal record of Cetotheriidae, *Cetotherium furlongi* (Kellogg, 1925) from the Early Miocene, requires taxonomical re-assessment, and its family affinities are unconfirmed (Steeman, 2007). *Metopocetus durinasus*, earlier dated as Langhian, was recently re-dated as Late Miocene (Marx, Bosselaers & Louwye, 2016). Therefore, up to now, the earliest records of the family are *Tiucetus rosae*, presumably from the Serravallian or Tortonian of Peru (13.8–9 Ma, Marx, Lambert & De Muizon, 2017), Herpetocetinae indet. from the Santa Margarita Fm. of California (12–10 Ma, Boessenecker, 2011) and *Zygiocetus nartorum* from the Krasnooktyabrskaya Fm., early Bessarabian (= late Serravallian, 12.1–11.6 Ma) of Maikop in Adygea, east to the Black Sea (Tarasenko, 2014). Another relevant specimen was identified as *Cetotherium* aff. *rathkei* and dates to the Badenian, Middle Miocene, of Bosnia (Stefanović, 2010; see below). The region of Paratethys where the latter two come from shows a great diversity of cetotheriids, based on reported findings (Gol’din & Startsev, 2017) starting with the nominative taxon of the family, *Cetotherium rathkii* (Brandt, 1843). This suggests that the earliest cetotheriids may have lived in that region.

Here a new baleen whale genus is described from the Middle Miocene of the Paratethys which is the chronologically earliest known record of Cetotheriidae in the world and the earliest diverging member of the family. Also, new data on taxa closely related to early cetotheriids are introduced and discussed here.

## Materials and Methods

### Nomenclatural acts

The electronic version of this article in portable document format will represent a published work according to the International Commission on Zoological Nomenclature (ICZN), and hence the new names contained in the electronic version are effectively published under that Code from the electronic edition alone. This published work and the nomenclatural acts it contains have been registered in ZooBank, the online registration system for the ICZN. The ZooBank LSIDs (Life Science Identifiers) can be resolved and the associated information viewed through any standard web browser by appending the LSID to the prefix http://zoobank.org/. The LSID for this publication is: urn:lsid:zoobank.org:pub:DD10D3E5-EE76-4198-B685-0892C18CB071. The online version of this work is archived and available from the following digital repositories: PeerJ, PubMed Central, and CLOCKSS.

### Specimens

**Figure 1:**
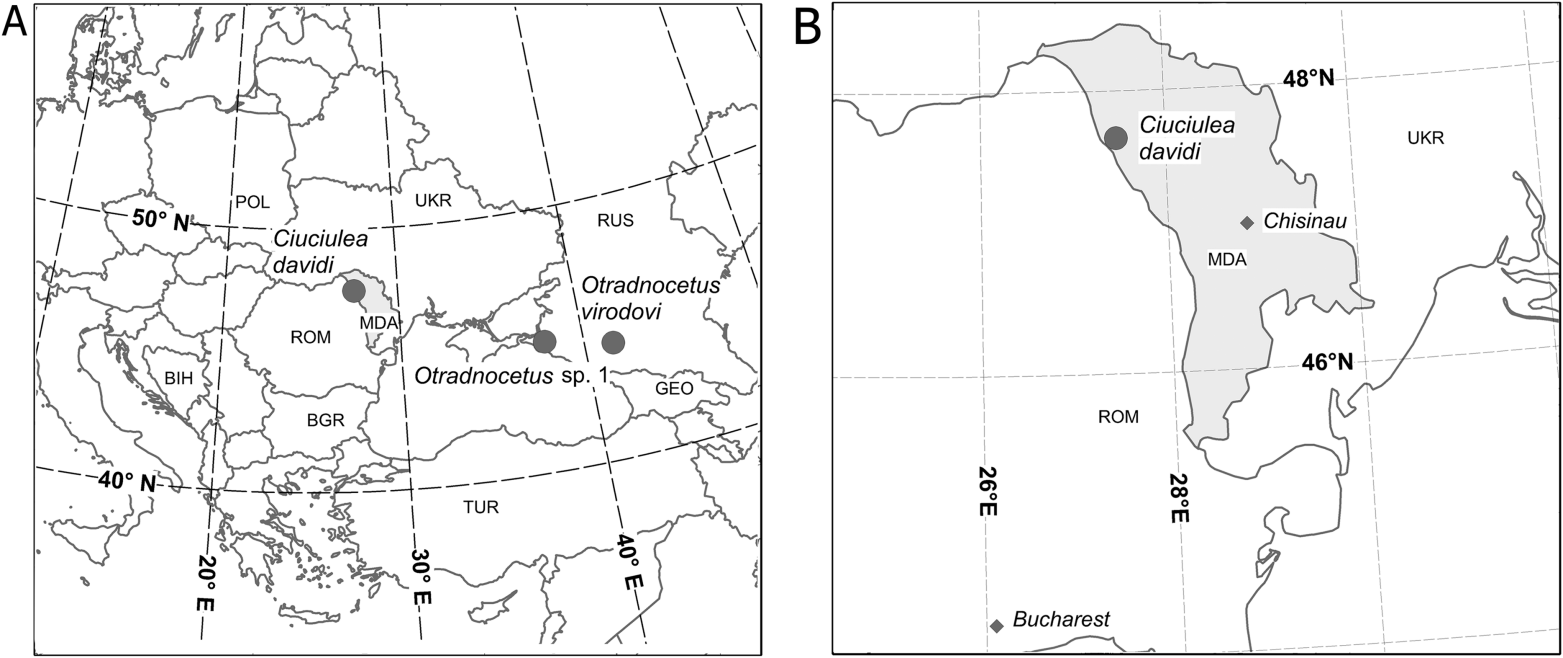
Geographical position of localities of *Ciuciulea davidi* (Moldova) and *Otradnocetus* spp. (Russia). (A) Europe; (B) the type locality of *Ciuciulea davidi* in Moldova.

The specimen ZIRM V 28/1, collected near the village *Ciuciulea* in the Republic of Moldova (Fig. 1), is housed by the Museum of Fossil Faunistic Complexes of Moldova, Institute of Zoology of Academy of Sciences of Republic of Moldova, Chisinău, Moldova.

The specimen GNM CO 1–90, collected near Otradnaya or Urupsky in the northwestern Caucasus (Fig. 1), is housed by the Georgian National Museum, Tbilisi, Georgia.

The specimen VSEGEI 2401, collected near the settlement Kievskoye, in the valley of the Kudako river in the northwestern Caucasus (Fig. 1), is housed by the Central Scientific Research Geological Survey Museum, Karpinsky Russian Geological Research Institute, St Petersburg, Russia.

Other specimens used for the phylogenetic analysis were earlier reported in the publications by Gol’din & Steeman (2015) and Gol’din & Startsev (2017).

### Measurements and analysis

Linear measurements were taken from the left side of the skeletons, and in some cases the right side was also measured (as indicated in tables); the lists of measurements for each specimen are provided in Table 1; Tables S2 and S3. Measurements were defined in the following way (unless otherwise indicated): length of the cranial, mandibular, and vertebral elements means anteroposterior distance, width means transverse distance, and height means vertical distance in relation to the longitudinal axis or sagittal plane of the body. For tympanic bulla and limb measurements length definitions are used in relation to the longitudinal axis of the bulla or the limb; limb width measurements are taken along the anatomically anteroposterior axis of the limb. Body size of *Otradnocetus* spp. was estimated from direct measurements of skeletons preserved in situ or in collection and after Lambert et al. (2010); body size category of *Ciuciulea davidi* was estimated in comparison with *Cetotherium riabinini*, for which the full skeleton was described (Gol’din, Startsev & Krakhmalnaya, 2014). The anatomical terminology generally follows Mead & Fordyce (2009) and Marx, Lambert & Uhen (2016a). The maps were compiled from the dataset by Sandvik (2009).

**Table 1:** Measurements (mm) of *Ciuciulea davidi*.

Measurement	Distance (mm)
Length of neurocranium (measured from the transverse line joining the antorbital notches to the occipital condyle)	285
Width between antorbital notches	175e
Length of nasal	74
Distal width of nasal	7
Greatest width of nares	32+
Distance between posterior margin of the nasal and anteriormost point of occipital shield	65
Anteroposterior length of parietal exposure on skull vertex	40
Minimum intertemporal width	100
Distance between posteriormost points of the paroccipital processes	182
Point-to-point distance between the dorsal margin of foramen magnum and anteriormost point of occipital shield	140
Maximum distance between outer margins of nuchal crests	180
Width of foramen magnum	47
Height of foramen magnum	43
Bicondylar width	89
Condylar height	66
Distance between medial margins of the foramina pseudovale	133
Distance between lateral margins of the basioccipital crests	73
Greatest height of neurocranium	136
Length of pars cochlearis	29
Height of pars cochlearis	11
Length of tympanic bulla	62
Width of tympanic bulla in ventral view	39
Length of humerus	152
Proximal width of humerus	62
Distal width of humerus	58
Centrum length/width/height, vertebra C6	17/60/50
Centrum length/width/height, vertebra C7	17/64/50
Centrum length/width/height, vertebra T1	18/60/40e
Centrum length/width/height, vertebra T2	23/59/35e
Centrum length/width/height, vertebra T3	27/60/37e
Centrum length/width/height, vertebra T4	37/60/40e
Centrum length/width/height, vertebra T5	38/60/44
Centrum length/width/height, vertebra T6	40/64/46
Centrum length/width/height, vertebra T7	46/62/47
Centrum length/width/height, vertebra T8	48/66/48
Centrum length/width/height, vertebra T9	48/68/47
Centrum length/width/height, vertebra T10	54/65/46
Centrum length/width/height, vertebra T11	57/69/47
Centrum length/width/height, vertebra L1	61/62/50
Centrum length/width/height, vertebra L2	62/62/52
Centrum length/width/height, vertebra L3	61/62/52
Centrum length/width/height, vertebra L4	64/62/52
Centrum length/width/height, vertebra L5	63/64/50

**Note:**

e, estimated value.

For the phylogenetic analysis, the heuristic parsimony analysis was conducted using TNT 1.1 (Goloboff, Farris & Nixon, 2008), “traditional search option,” 10,000 replicates, tree bisection-reconnection branch swapping, saving 10 trees per replicate, based on the earlier published matrix (Gol’din & Startsev, 2017) with the relevant updates concerning recently described *Tiucetus rosae* (Marx, Lambert & De Muizon, 2017) and hereby introduced, new taxa, *Ciuciulea davidi* (ZIRM V 28/1) and *Otradnocetus* sp. (VSEGEI 2401) (the full character-taxon matrix is provided in the Table S1):

*Ciuciulea davidi*           -11????1110 212?12???? 11?000?0?? ? ?????1110 0?1 11?1?0 0 11??2??1?? 1?? ?? 100000 001012 ??????????? ?0?? 0???1021??
*Tiucetus rosae*         -1110??1100 111?00???0 1110101100 0 111001120 101 1??110 0 000?22?1?? 1?? 00 120000 000002 ??????????? ???? ??????????
*Otradnocetus* sp.          -?????????? ??????0100 1????????? 0 1110????? ??? 1????? ? ?????????? ??? ?? 10?0?? ??00?0 12111211101 ?01? ???211210?

A strict consensus tree was obtained from the resulting eight most parsimonious trees. Branch support was estimated using symmetric re-sampling (change probability = 33), 2,000 replicates, as frequency differences, and branch support values were presented as GC scores.

## Results

### Geological setting

The specimen ZIRM V28/1 was collected in 1965 near the village *Ciuciulea* in the Republic of Moldova (approximate geographic coordinates 47°40′N, 27°28′E) (Fig. 1) by Anatolie David. It was recovered from the late Badenian outcrops of the Fore-Carpathian Basin of the Paratethys (= early Serravallian, between 13.82 and 12.82 or 12.65 Ma), Middle Miocene. In the original field documentation Badenian is indicated as the age of the specimen, and the late Badenian is the most likely age of the outcrops in the area (Popov et al., 2004). This was possibly also the age of the easternmost expansion of the Fore-Carpathian Basin (Rögl, 1998). The end of this stage is dated as 12.82 Ma (Hír et al., 2016) or 12.65 Ma (Palcu et al., 2015) and characterized by a salinity crisis and mass extinction of biota (Wysocka et al., 2016): this provides a well-constrained upper age boundary for the specimen. The specimen was taken from a facies of siltstones with high clay content and without gypsum.

The specimen VSEGEI 2401 was collected in 1927 from an exploratory pit for the oil production near the settlement Kievskoye, in the valley of the Kudako river in the northwestern Caucasus (approximate geographical coordinates 45°00′N, 37°53′E) (Fig. 1) by A. N. Fedorov and described by Riabinin (1934, 1937). It was found in the late Sarmatian s.l. deposits of the Eastern Paratethys (= Tortonian, between 10 and 7.5 Ma, as by Radionova et al., 2012; Popov et al., 2013), Late Miocene. It was found in a 0.65 m thick layer of greenish green clay with dispersed gypsum and fish remains, covered by two m thick re-worked alluvium of Sarmatian origin. Its age was identified by Riabinin (1934 and refences therein), based on presence of a bivalve *Mactra caspia* in the clay layer (as confirmed by modern stratigraphy: see Vernyhorova, 2015).

## Systematic Paleontology

**Figure 2:**
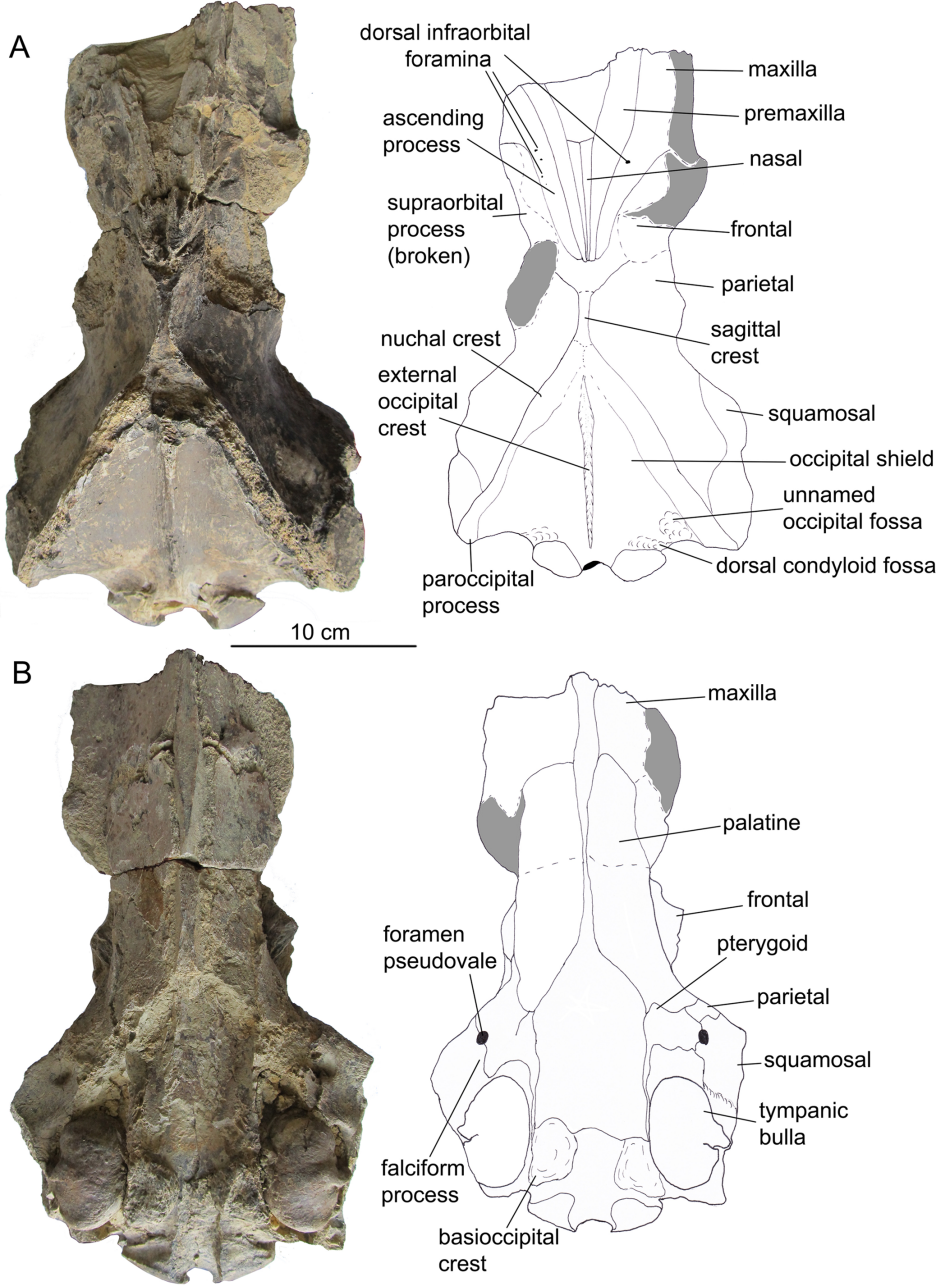
The skull of *Ciuciulea davidi*, ZIRM V 28/1 (holotype), from the Middle Miocene of Moldova. (A) Dorsal view; (B) ventral view. The scale bars equal 10 cm.

**Figure 3:**
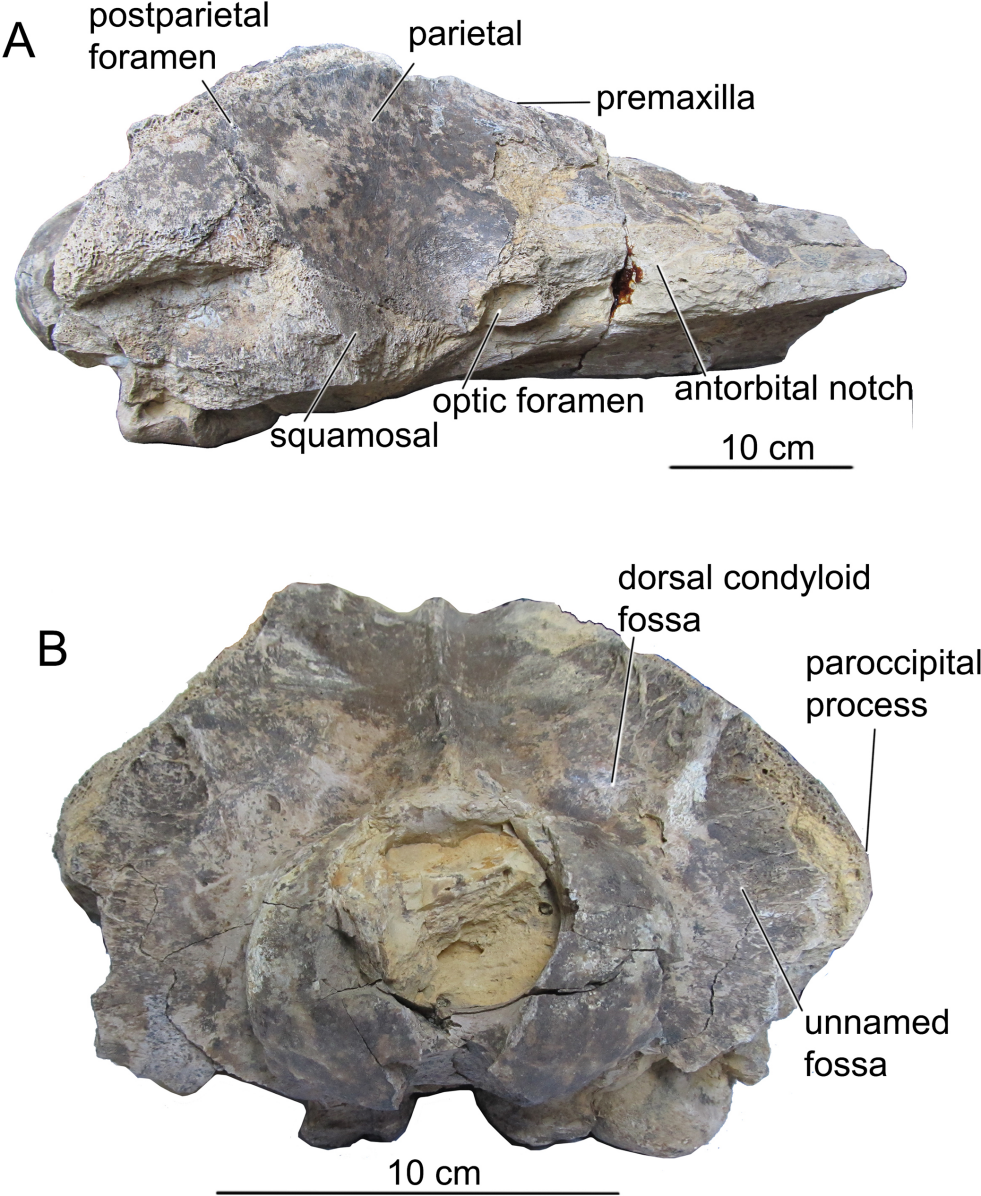
The skull of *Ciuciulea davidi*, ZIRM V 28/1, from the Middle Miocene of Moldova (holotype) (cont.). (A) Lateral view; (B) posterior view. The scale bars equal 10 cm.

**Figure 4:**
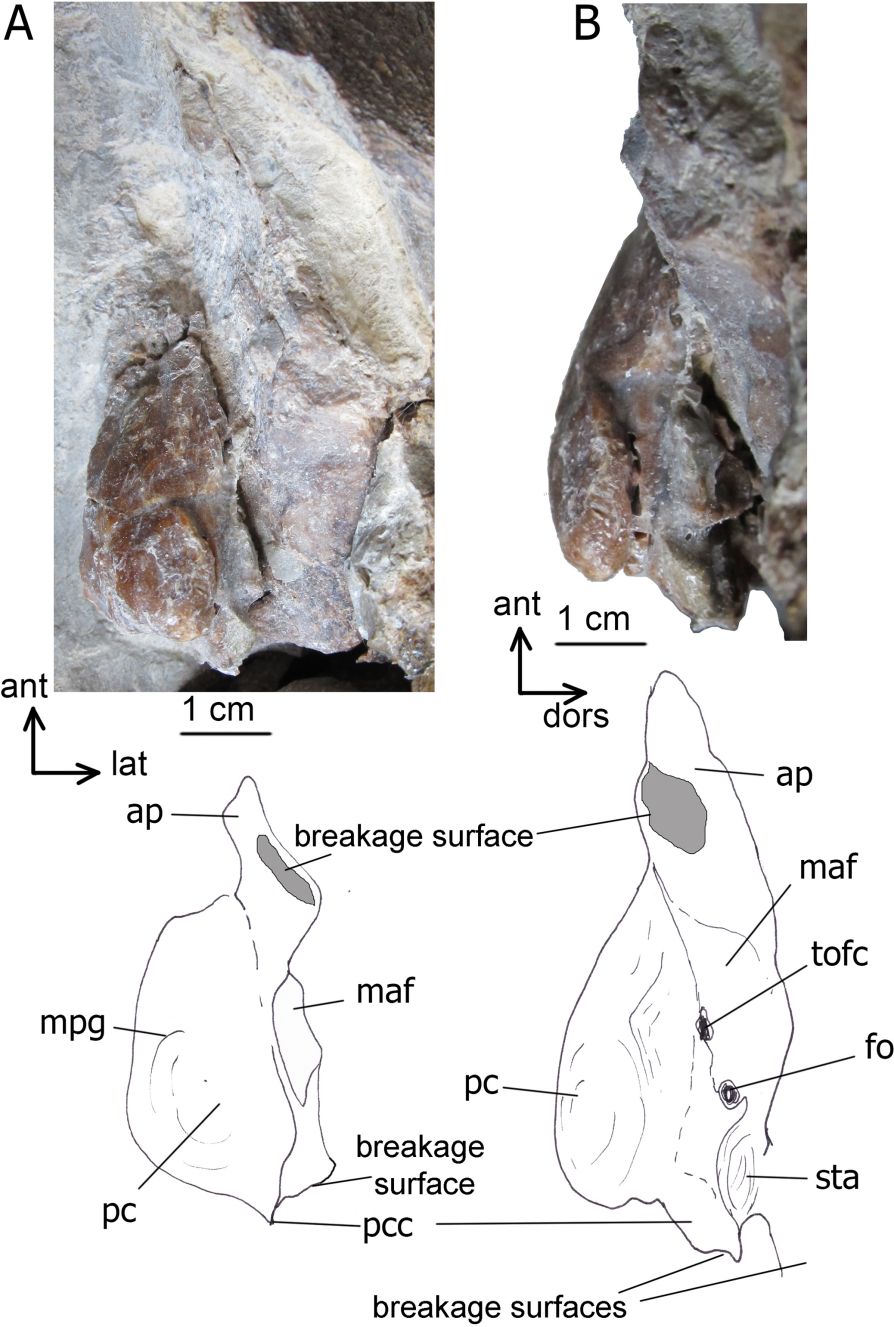
The left periotic bone of *Ciuciulea davidi*, ZIRM V 28/1 (holotype). (A) Ventral view; (B) lateral view. Abbreviations: ant, anterior; ap, anterior process; dors, dorsal; fo, fenestra ovalis; lat, lateral; maf, mallear fossa; mpg, median promontorial groove; pc, pars cochlearis; pcc, posterior cochlear crest; sta, stapedial fossa; tofc, tympanic opening of the facial canal. The identical scale bars equal one cm.

**Figure 5:**
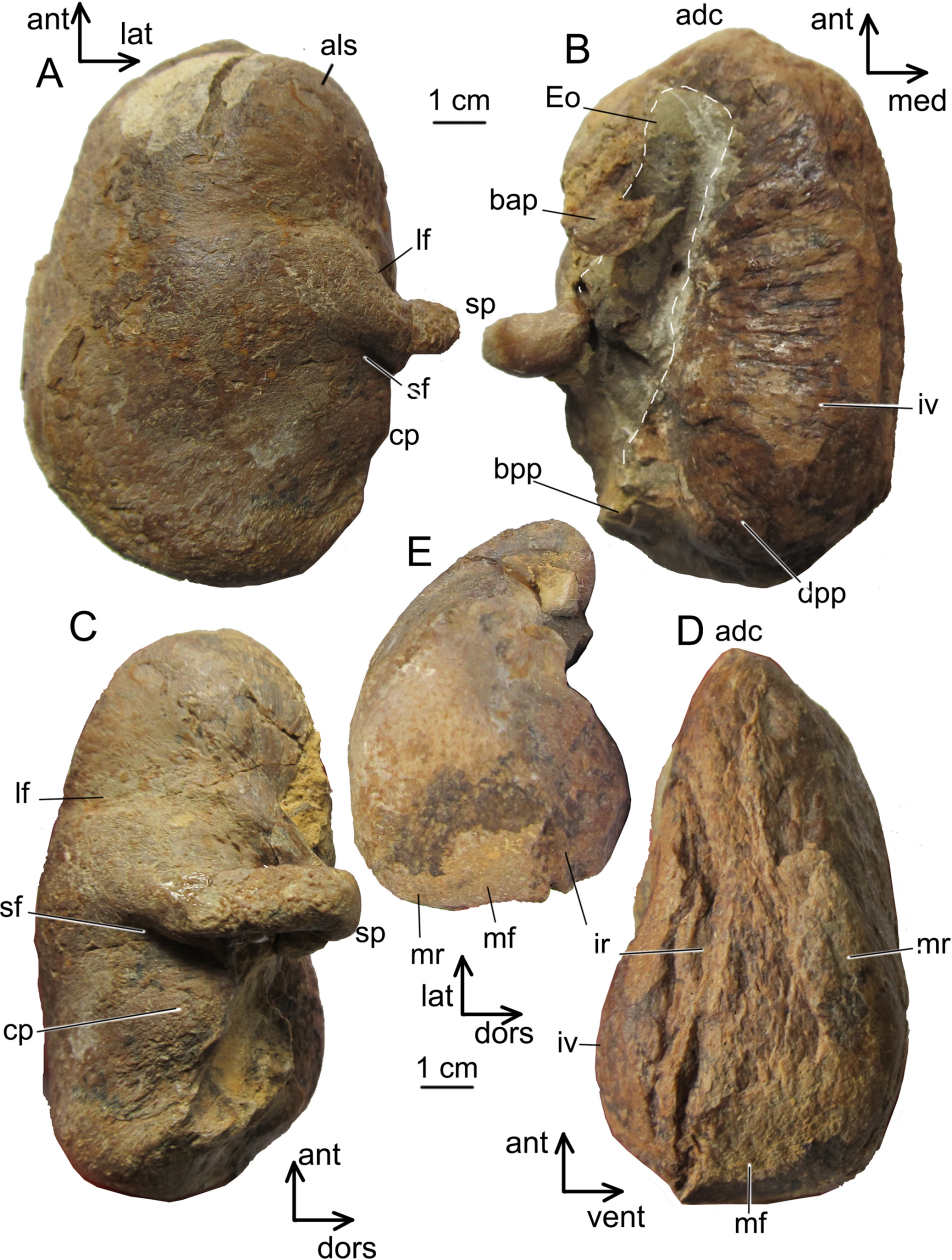
The left tympanic bulla of *Ciuciulea davidi*, ZIRM V 28/1 (holotype). (A) Ventral view; (B) dorsal view; (C) lateral view; (D) medial view, (E) posterior view. Abbreviations: adc, anterodorsal crest; als, anterolateral shelf; ant, anterior; bap, bulla anterior pedicle; bpp, bulla posterior pedicle; cp, conical process; dors, dorsal; dpp, dorsal posterior prominence; Eu, Eustachian outlet; ir, involucral ridge; iv, involucrum; lat, lateral; lf, lateral furrow; med, medial; mf, medial furrow; mr, main ridge; sf, groove posterior to the sigmoid process (“sigmoid fissure”); sp, sigmoid process; vent, ventral. The scale bars equal one cm.

**Figure 6:**
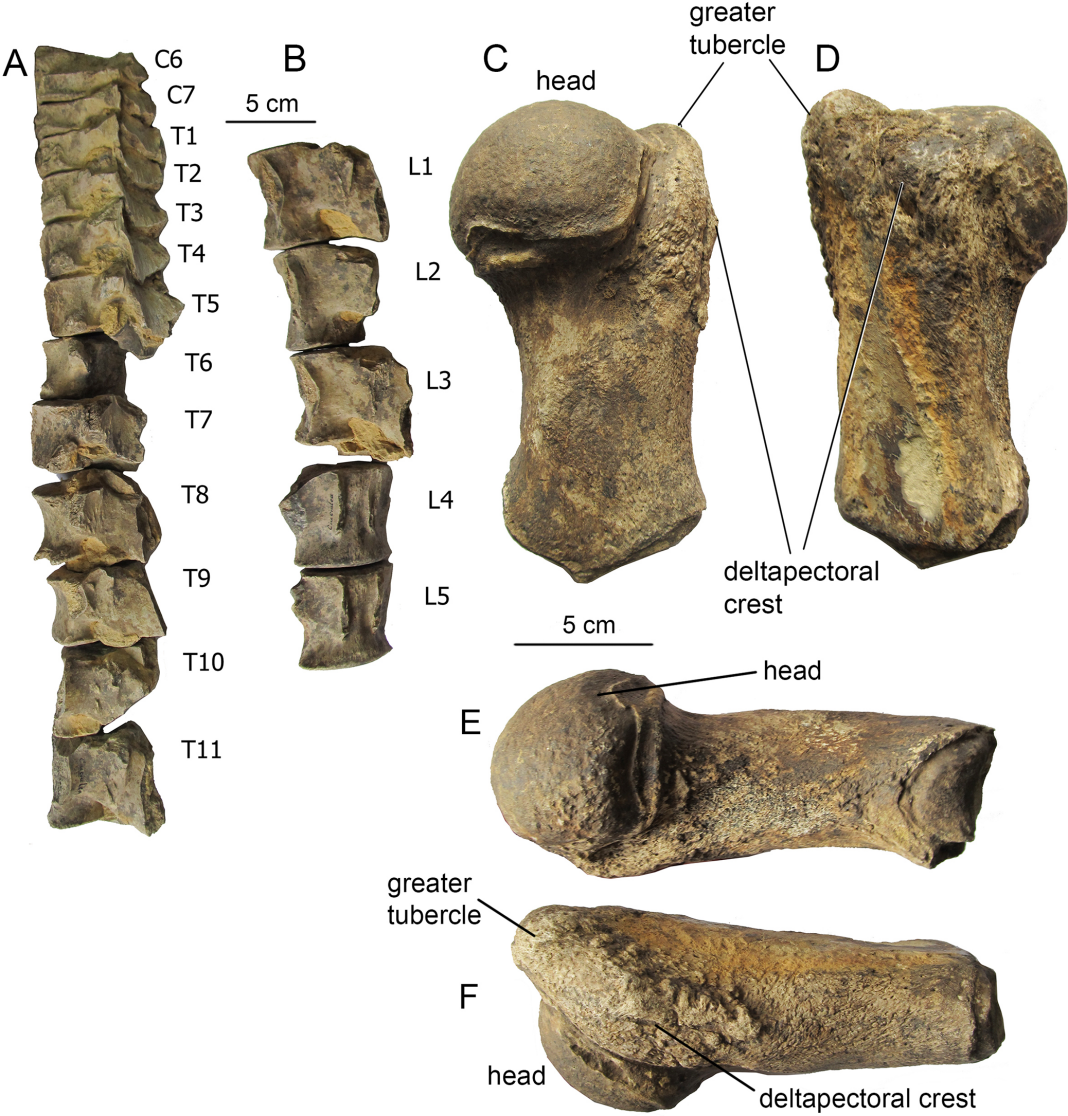
Postcranial skeleton elements of *Ciuciulea davidi*, ZIRM V 28/1 (holotype). (A) Cervical and thoracic vertebrae, left lateral view; (B) lumbar vertebrae, left dorsolateral view; (C) right humerus, lateral view; (D) right humerus, medial view; (E) right humerus, posterior view; (F) right humerus, anterior view. The scale bars equal five cm.

CETACEA Brisson, 1762
MYSTICETI Flower, 1864
**PLICOGULAE** Geisler et al., 2011
CETOTHERIOIDEA sensu Gol’din & Steeman, 2015
CETOTHERIIDAE Brandt, 1872; sensu Gol’din, Startsev & Krakhmalnaya, 2014
*CIUCIULEA DAVIDI*, gen. et sp. nov.
urn:lsid:zoobank.org:act:3DC65FC9-DA74-4D13-9104-4D4F03742883
urn:lsid:zoobank.org:act:A4E90DA0-AF0E-48B5-BA81-6E54CB181D33
(Figs. 2–6 and Table 1)

**Holotype.** ZIRM V 28/1, an incomplete skull with periotics and tympanic bullae (the left of which was detached for description), lacking rostrum, orbits and most of squamosals; 18 partial vertebrae; a fragment of the right scapula and a right humerus.

**Diagnosis.** A small-sized whale approximately three to four m long differing from all the other Cetotheriidae in the presence of a narrow occipital shield, which is as long as wide; and a pars cochlearis of the periotic bone bulging out ventral to fenestra rotunda (a plesiomorphic state for the family). The species retains a primitive state among Cetotheriidae in that the premaxillae form a transverse line with the posterior ends of nasals and maxillae rather than constricted or overridden by ascending processes of maxillae. Differs from *Otradnocetus*, *Parietobalaena*, *Diorocetus*, and *Tiucetus* in strongly telescoped wedge-shaped facial bones, absence of a squamosal cleft, and a tympanic bulla anteriorly narrowing from the medial view, with a shallow lateral furrow, and a short anterior lobe; differs also from *Parietobalaena* in a transversely short sigmoid process of the tympanic. Differs from Tranatocetidae in rostral bones not overriding frontals, a flat occipital shield, and a tympanic bulla with a posterior portion not swollen in the posteroventral area, a shallow lateral furrow, and a low conical process. Differs from all the other Cetotheriidae except *Joumocetus shimizui* in a relatively long interparietal region bearing a sagittal crest. Differs from all the other Cetotheriidae except *Z. nartorum* in a transversely narrow pars cochlearis with a short posterior cochlear crest. Differs from all the other Cetotheriidae except *Metopocetus* spp. in a reduced lateral projection of anterior process of the periotic bone. Differs from all the other Cetotheriidae except *Mithridatocetus* spp. in a poorly developed groove for tensor tympani muscle on the periotic bone. Differs also from *J. shimizui* in an occipital shield protruding slightly anterior to the center of the temporal fossa; differs from *Z. nartorum* in a triangular occipital shield with straight (rather than bowed) lateral margins and a tympanic bulla narrower anteriorly than posteriorly in ventral view.

**Locality and age.** *Ciuciulea* in the northern Moldova, the late Badenian (13.82–12.65 Ma) of the Fore-Carpathian Basin of the Paratethys, late Middle Miocene.

**Derivation of name.** The genus name (English spelling: [ʧuʧuːlɪə], Ch-u-ch-u-l-i-a) derives from the type location. The species is named after its collector, Dr. Anatolie David.

### Description

**Ontogenetic age.** All the vertebral epiphyses are fully fused with centra. The sutures are mostly obliterated, and the suture lines can be traced only in the anterior lumbar vertebrae. Also, the epiphyses are fully fused in the humerus. Interestingly, many sutures of the braincase also attain an advanced degree of obliteration: no traces of sutures between sphenoid bones, alisphenoids or presphenoids, and between basisphenoid and basioccipital are seen, and parietal-squamosal sutures are only barely visible. Therefore, the holotype specimen is considered to be a physically mature, fully grown individual with a particularly high degree of skeletal ossification, that is, unusual among the edentulous mysticetes.

**Cranium.** The cranial fragment is the neurocranium with preserved posterior portions of the rostral bones, with associated periotics and tympanic bullae, lacking the zygomatic processes of squamosals (Figs. 2 and 3). The **neurocranium** is relatively narrow and low (Table 1): the braincase height of 136 mm is among the smallest for Neogene chaeomysticetes, comparable only with *Parietobalaena*, *Cetotherium*, and *Joumocetus*. Rostral bones are deeply wedged posterior above the frontals, and they presumably reach the level of posterior portions of the orbits, anterior to the center of the temporal fossa; this is a distinct feature of Cetotheriidae, as well as *Uranocetus*, *Titanocetus*, Eschrichtiidae, and Balaenopteridae. Inferences about the base of the rostrum can only be made on the basis of the outlines of ascending processes of the maxillae. The **premaxilla** is not covered by adjoining bones and is exposed on the skull surface on all its extension in dorsal view. It gradually tapers on its posterior end and terminates at the same level as the posterior margin of the nasal, forming a transverse line with the posterior ends of nasals and maxillae and separating the nasal and the maxilla along their entire length. The ascending process of the **maxilla** tapers toward its apex, and its lateral margin is posterolaterally concave in dorsal view, as in the other Cetotheriidae. The apices of the processes approximate each other but do not converge; the angle between their lateral margins is 45° (Fig. 2A). At least, four small dorsal infraorbital foramina are seen on the left side, and one foramen of the moderate size (possibly, the primary dorsal infraorbital foramen in terminology by Marx, Bosselaers & Louwye, 2016) is situated on the right side, posterior to the base of rostrum. A few more foramina are seen on palatine process near the anterior margin of the palatine bone on the ventral side of the skull. The **nasal**, as preserved, is a long triangular bone with the anterior margin slightly anterior to the base of the rostrum. Its anterolateral portion protrudes slightly anterior to the anteromedial one. The **frontal** is narrowly exposed posterior to the ascending process of the maxilla (Fig. 2A). The supraorbital process, as preserved at its base, is anterolaterally directed in dorsal view and gradually sloping in anterior view. The frontal-parietal suture forms an anteriorly directed obtuse angle in lateral view; it is obliterated on the right side.

The intertemporal region of the **parietals** is anteroposteriorly long, nearly 40 mm, and widely exposed on the vertex between the facial bones and occipital shield (Fig. 2A). It bears a sagittal crest, which is preserved only as the base. There is no separate interparietal bone. The medial surface of the temporal fossa is concave in dorsal view, making the braincase particularly narrow. Numerous small foramina with associated nearly vertical sulci are scattered on this surface. The parietal-squamosal suture is anteroventrally directed in lateral view (Fig. 3A); it contacts the occipital shield in its posterior portion. It is neither keeled nor bulged into the temporal fossa. A small but distinct postparietal foramen is seen on the suture just ventral to the occipital shield. This suture is well-fused, similar to all the sutures of the braincase. It is barely visible on the outer surface of the skull. The postparietal foramen is not associated with the alisphenoid, unlike in *Herpetocetus morrowi* (El Adli, Deméré & Boessenecker, 2014). For the anatomy of the parietal region *Ciuciulea* seems to be primitive among Neogene mysticetes and resembles *Parietobalaena*: however, the cetotheriid *Joumocetus* is quite similar to it, and so are *Pinocetus* and *Cephalotropis*. The latter two taxa are here considered as members of a cetotherioid clade sister group to Cetotheriidae (Gol’din & Steeman, 2015).

The anterior margin of the **palatine** is situated anterior to the presumed base of the rostrum (Fig. 2B). It is notched in ventral view, with a distinct anteriorly protruding anteromedial lobe. The palatines do not converge along the midline of the rostrum, and their anteromedial margins form together a V-shaped notch bordering a vomer window (similar to *Cetotherium riabinini*: Gol’din, Startsev & Krakhmalnaya, 2014). The posterior margin of the palatine is situated at the level of the center of the temporal fossa, well anterior to the foramen pseudovale. The **pterygoid** is widely exposed on the ventral side of the skull between the squamosal, the palatine, and the parietal (Fig. 2B). The pterygoid sinus fossa anteriorly extends until the level of the foramen pseudovale. The medial lamina borders the vomer medially; its posterior margin is anterior to the basioccipital crest. The posterolateral margin of the lateral lamina of the pterygoid borders the squamosal and forms the medial portion of the foramen pseudovale. The ventral lamina and pterygoid hamulus have not been preserved. The **vomer** forms a long rhomboid window between the palatines and maxillae on the ventral side of the rostrum. The posterior margin is damaged. The position of the trace of the vomer on the ventral surface of the skull suggests that the vomer does not reach basioccipital crests and forms a transverse line with posterior margins of pterygoids. The **basisphenoid** is partly exposed on the ventral side posterior to the vomer and dorsally to it where the vomer is broken. Zygomatic processes of the **squamosals** have not been preserved, as well as most of the rest of the squamosals. The squamosal plate is relatively small, and it occupies only the posterior portion of the temporal fossa. No squamosal cleft is observed. The falciform process is short, robust and rhomboid.

The **exoccipital** is completely fused with the supraoccipital, and the suture preserved as a distinct crest with the traces of muscle attachment on it (Figs. 2A and 3B). Both the dorsal and ventral condyloid fossae are well pronounced. Another unnamed shallow fossa is situated lateral from the occipital condyle for attachment of the rectus capitis posterior major or rectus capitis lateralis muscle (Murie, 1873). The paroccipital process, as preserved, is anteroposteriorly short and transversely thin. The distinct paroccipital concavity is partly preserved on its ventral side. The foramen magnum is sub-circular, slightly wider than high. Occipital condyles are wide and dorsoventrally symmetrical; they approximate each other neither ventral, nor dorsal to the foramen magnum. Each condyle has a pronounced neck, and the overall condylar area is anteroposteriorly elongated (Fig. 3B).

The anteriormost point of the **occipital shield** is at the level of the center of the temporal fossa (Fig. 2A). In dorsal view, the occipital shield is equilaterally triangular. It is significantly narrower than in the other Cetotheriidae: its width to height ratio is only 1.29, whereas in other members of the family it is within 1.48–2.35 (Bouetel & De Muizon, 2006; Gol’din, Startsev & Krakhmalnaya, 2014). The external occipital crest is low but distinct, with broad and shallow unnamed fossae for attachment of the semispinalis capitis muscle. The **basioccipital** is completely fused with the adjoining bones. Sub-triangular basioccipital crests are wide and moderately high, with deep laterally situated fossae, probably for attachment of the longus capitis muscle (Marx, Bosselaers & Louwye, 2016). *Ciuciulea* is distinct among Neogene baleen whales for the great diversity of fossae for attachment of various muscles on the occipital bones.

**Periotic.** In ventral view, the anterior process is short and transversely compressed; in lateral view, it is rhomboid (Fig. 4). Its lateral projection is indistinct, seen as small tubercle. The oval mallear fossa is large and shallow. The sub-rectangular pars cochlearis is long, transversely narrow, dorsoventrally high and mostly smooth. Its anterior portion is bordered with irregularly shaped projections, the longest of which is the anteriormost one. The median promontorial groove is seen on it. The fenestra ovalis is anteroposteriorly long. The fenestra rotunda is large (four mm in diameter) and circular. The pars cochlearis is slightly protruded ventral to it. The posterior cochlear crest (= caudal tympanic process) is very short and rounded. It reaches the base of the posterior process as a broad and high crest. The stapedial fossa is broad and shallow. The facial sulcus is shallow in the preserved portion. The compound posterior process has not been preserved, except the base. In overall structure, the periotic bone of *Ciuciulea* is primitive, and it generally fits to the description of Diorocetidae sensu Steeman, 2007, or *Parietobalaena* (Kellogg, 1924; Steeman, 2010); it lacks, as seen from the ventral and posterior sides, some apomorphies of Cetotheriidae like the development of a lateral projection of the anterior process or a robust posterior cochlear crest.

**Tympanic bulla.** In ventral view, the tympanic bulla is box-shaped, with the low and wide main ridge and angular anterior margin, with oblique anterolateral and anteromedial edges. The anterolateral shelf is very shallow. In ventral view, the bulla is oval, with an angular anterior margin (Fig. 5A). The main ridge is oblique to the longitudinal axis of the bulla. The sigmoid process is transversely straight, short, and equally thickened anteroposteriorly: its main axis directed slightly posterolaterally, and the ventralmost portion is perpendicular to the longitudinal axis of the skull. Its slightly inflated base is at the level of the center of the bulla. The sigmoid fissure and lateral furrow are shallow. The conical process is extremely low and rounded; posterior to it, there is a shallow fossa. In dorsal view, the finely grooved involucrum is long and narrow, with a bulbous globular dorsal posterior prominence. The anterodorsal crest is high and sharp (Fig. 5B). In lateral view, the bulla is seen as box-shaped narrowing posteriorly and rounded anteriorly (Fig. 5C). In medial view, the bulla is sub-triangular, tapering anteriorly. The main and involucral ridges do not join together, and the distinct main ridge is extended as the anterodorsal crest (Fig. 5D). In posterior view, the bulla is roughly rhomboid (Fig. 5E). The bulla of *Ciuciulea* overall is of typical generalized cetotheriid appearance and resembles *Herpetocetus* or *Brandtocetus*, differing only in being transversely narrower: its length to width ratio is 1.6, similar to *Metopocetus hunteri* (Marx, Bosselaers & Louwye, 2016).

**Vertebral column.** Two cervical, 11 thoracic, and five lumbar vertebrae have been preserved. The seven anteriormost, the best preserved, are still attached with sediment to each other; from others, mostly the centra remain with broken processes. In contrast to Paratethyan cetotheres of the sub-family Cetotheriinae (Gol’din & Startsev, 2017), none of the vertebrae is pachyosteosclerotic. None of the vertebrae are fused with each other.

The sixth and the seventh cervical vertebrae, C6 and C7, have been preserved. In C6, a rudimentary parapophysis is present along with the diapophysis, and in C7 only a short diapophysis is developed. Eleven thoracic vertebrae seem to contain the consecutive series. Facets on the centra for the articulation with the rib capitula are present on the first seven thoracic vertebrae, corresponding to the chest functional unit (Buchholtz, 2001); this is consistent with the records from *Piscobalaena nana* (Bouetel & De Muizon, 2006) and *Cetotherium riabinini* (Gol’din, Startsev & Krakhmalnaya, 2014). The neural spines of the chest vertebrae are anterior deflected, whereas those of the more posterior thoracics are posterior deflected. Two posteriormost vertebrae (presumably, T10 and T11) are considerably longer than others, similar to a few other cetaceans, including *Caperea marginata* (Buchholtz, 2001, 2011). Lumbar vertebrae are similar to each other in size and proportions: the centra are as long as wide, oval in anterior view and slightly flattened dorsoventrally, with height to width ratios of 0.80–0.85.

**Scapula.** Only the glenoid portion has been preserved with a remnant of a small coracoid process and the base of the acromion.

**Humerus (Fig. 6A).** The diaphysis is dumbbell-shaped in lateral view. The humeral head is semi-globular and relatively small, with the diameter less than a third of the total length of the humerus. The head is shifted to the posterior surface of the diaphysis, and a short neck is seen. Both the greater and lesser tubercles are well-developed, short, and divided by a shallow furrow. This generalized anatomy is typical for a few Plicogulae mysticetes from the Middle Miocene in which the humerus is known, such as *Thinocetus, Otradnocetus*, or *Pinocetus*.

Another specimen is very similar to *Ciuciulea davidi* and likely represents the same taxon, at least at the genus level. This is a whale recorded by Stefanović (2010) from Štrbci in northern Bosnia, originally referred to as *Cetotherium* aff. *rathkei* and dated from the Badenian, with no additional sub-divisions. Its partially preserved tympanic bulla is identical to that of *Ciuciulea davidi* in medial view and quite similar in posterior view. Its humeral diaphysis is also similar to that of *Ciuciulea davidi*, although this is not a very diagnostic element. Finally, the vertebrae resemble *Ciuciulea davidi* in their external appearance, size and proportions. Thus, this specimen is hereby identified as *Ciuciulea* sp., and the range of the genus *Ciuciulea* includes the Fore-Carpathian basin and the Central Paratethys between modern Bosnia and Herzegovina and Moldova.

**Figure 7:**
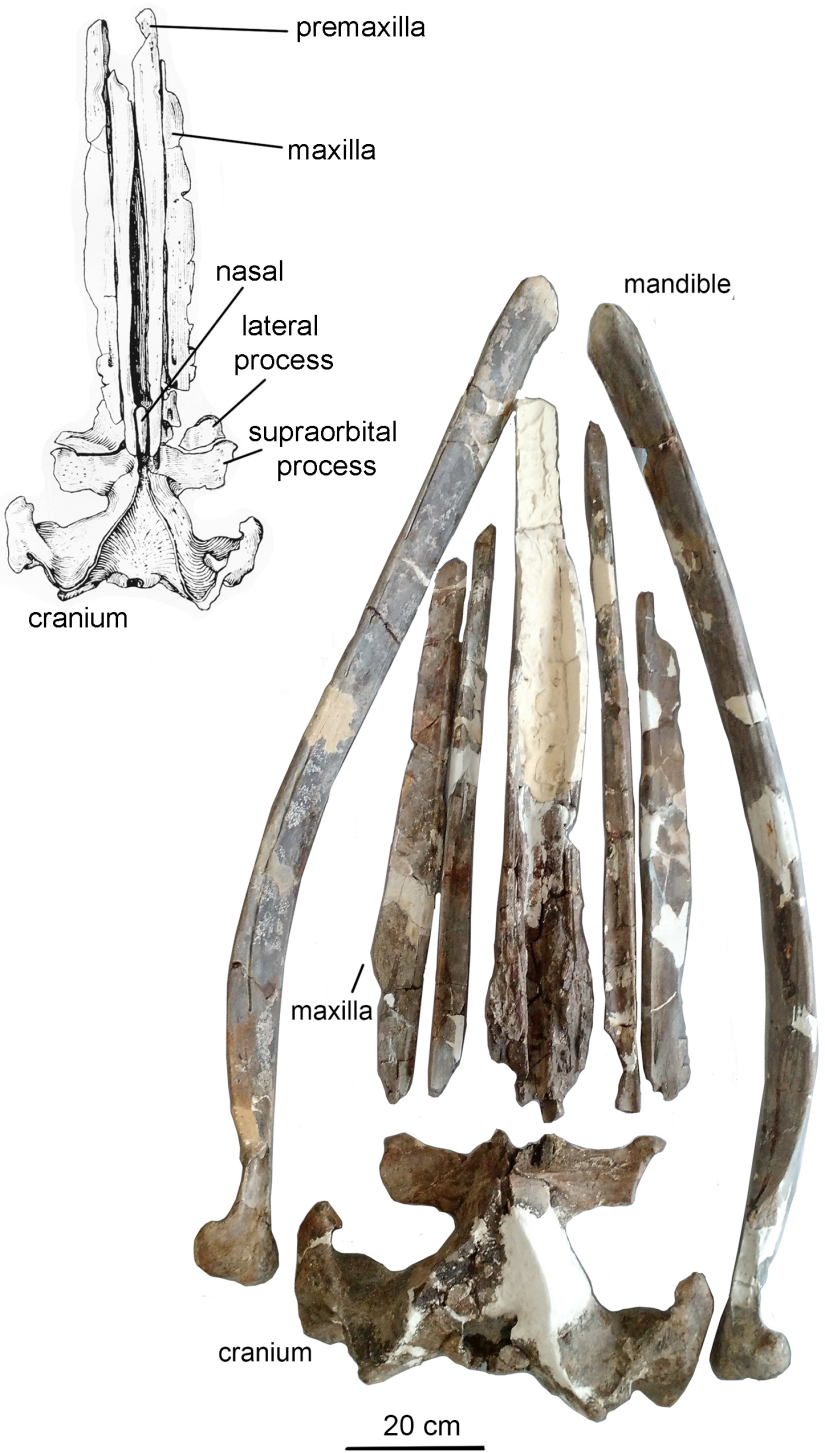
The skull and mandibles of *Otradnocetus virodovi*, GNM CO-1–3. General view, with a sketch drawing from the original description (Mchedlidze, 1984: 21). The scale bar equals 20 cm.

**Figure 8:**
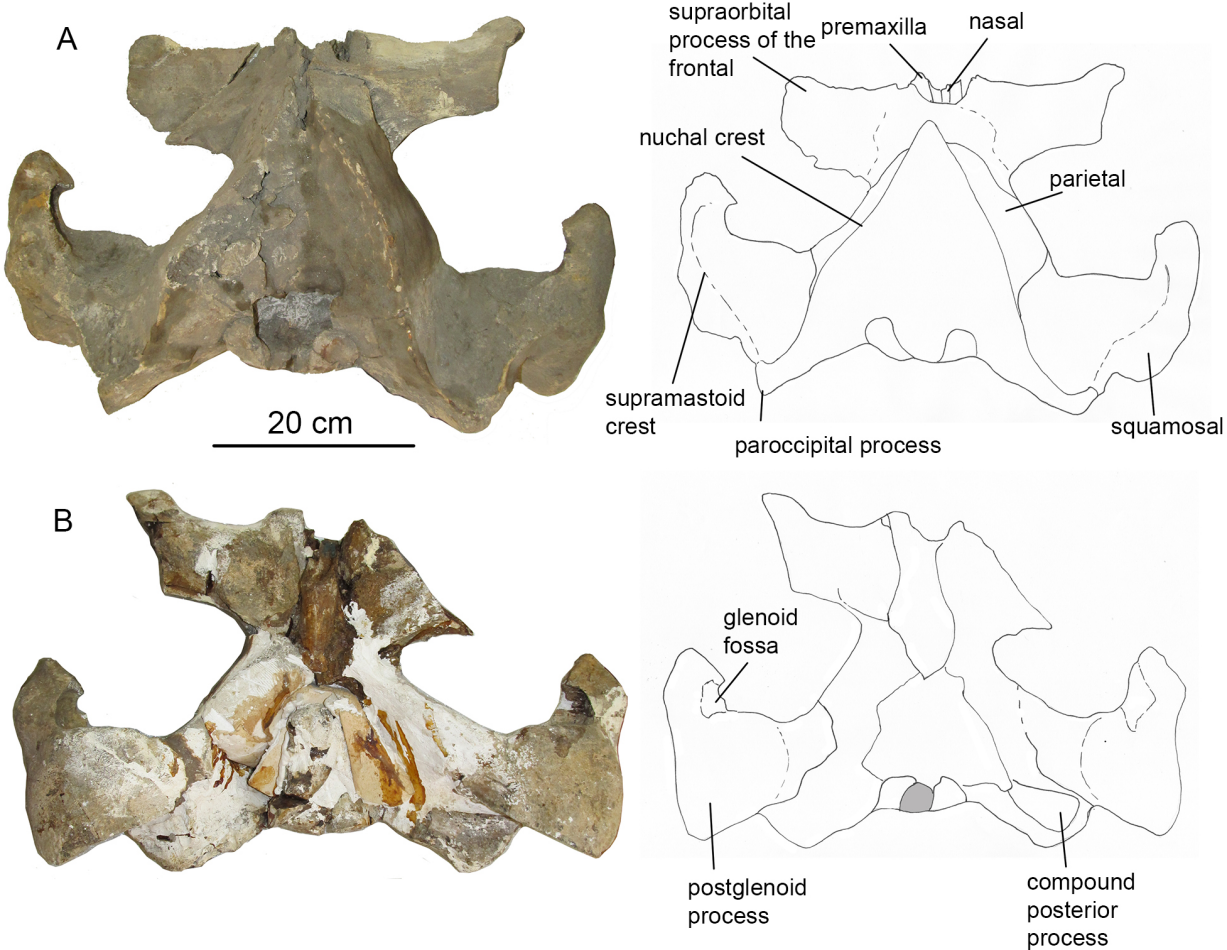
The skull of *O. virodovi*, GNM CO-1. (A) Dorsal view; (B) ventral view. The scale bar equals 20 cm.

**Figure 9:**
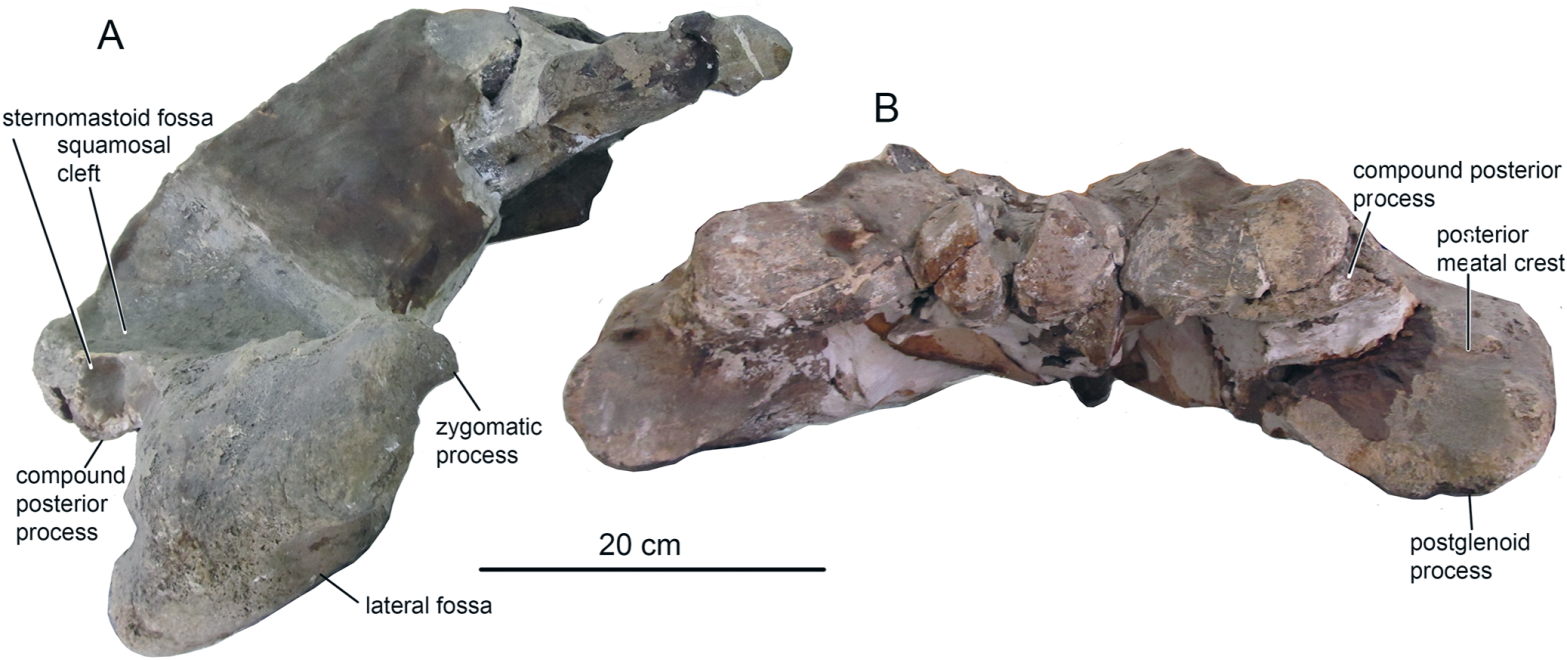
The skull of *O. virodovi*, GNM CO-1 (cont.). (A) Right lateral view; (B) posterior view. The scale bar equals 20 cm.

**Figure 10:**
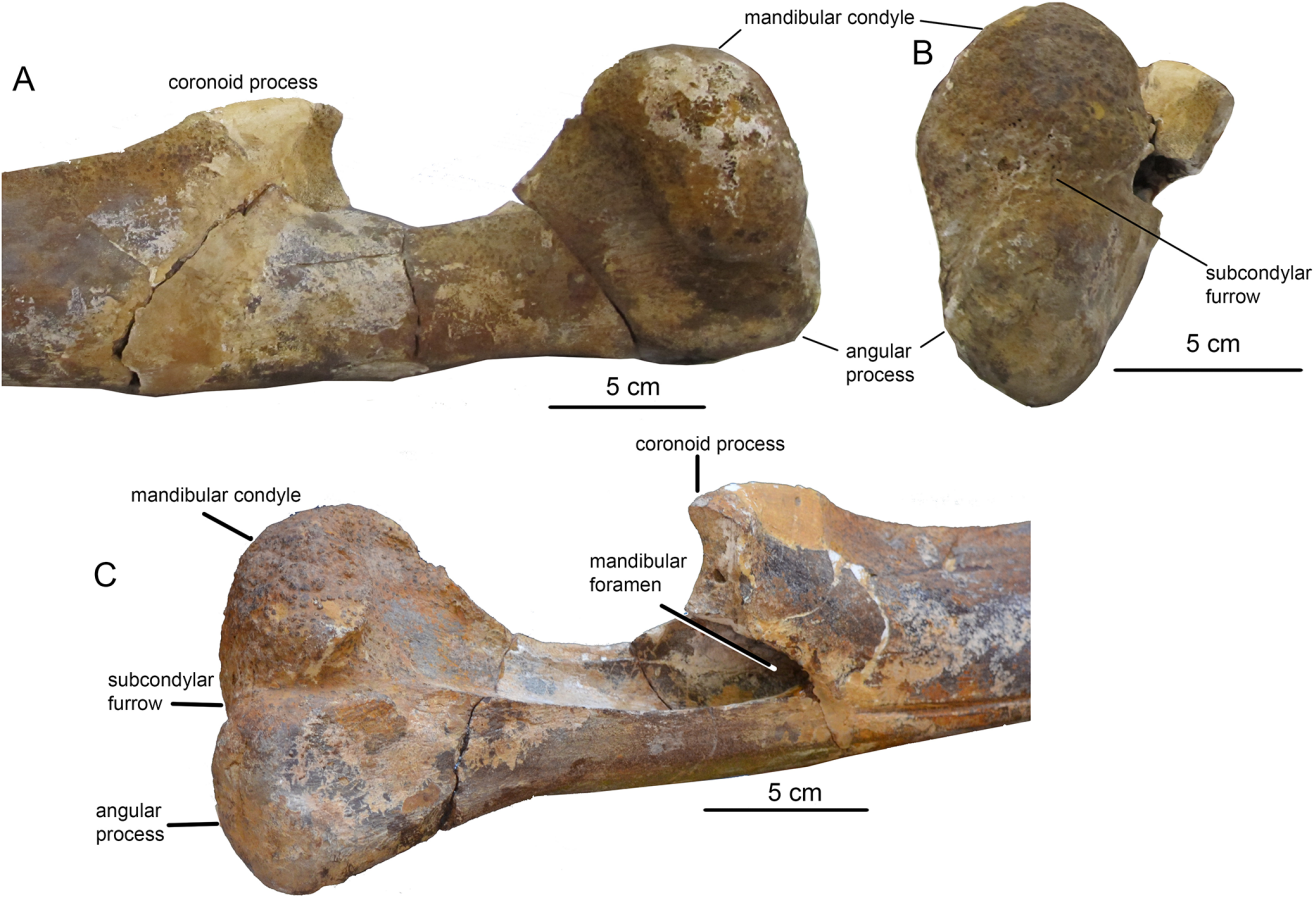
The left ramus of mandible of *O. virodovi*, GNM CO-3. (A) Posterior part, lateral view; (B) posterior view; (C) medial view. The scale bars equal five cm. Figure 10C courtesy of Maia Bukhsianidze.

**Figure 11:**
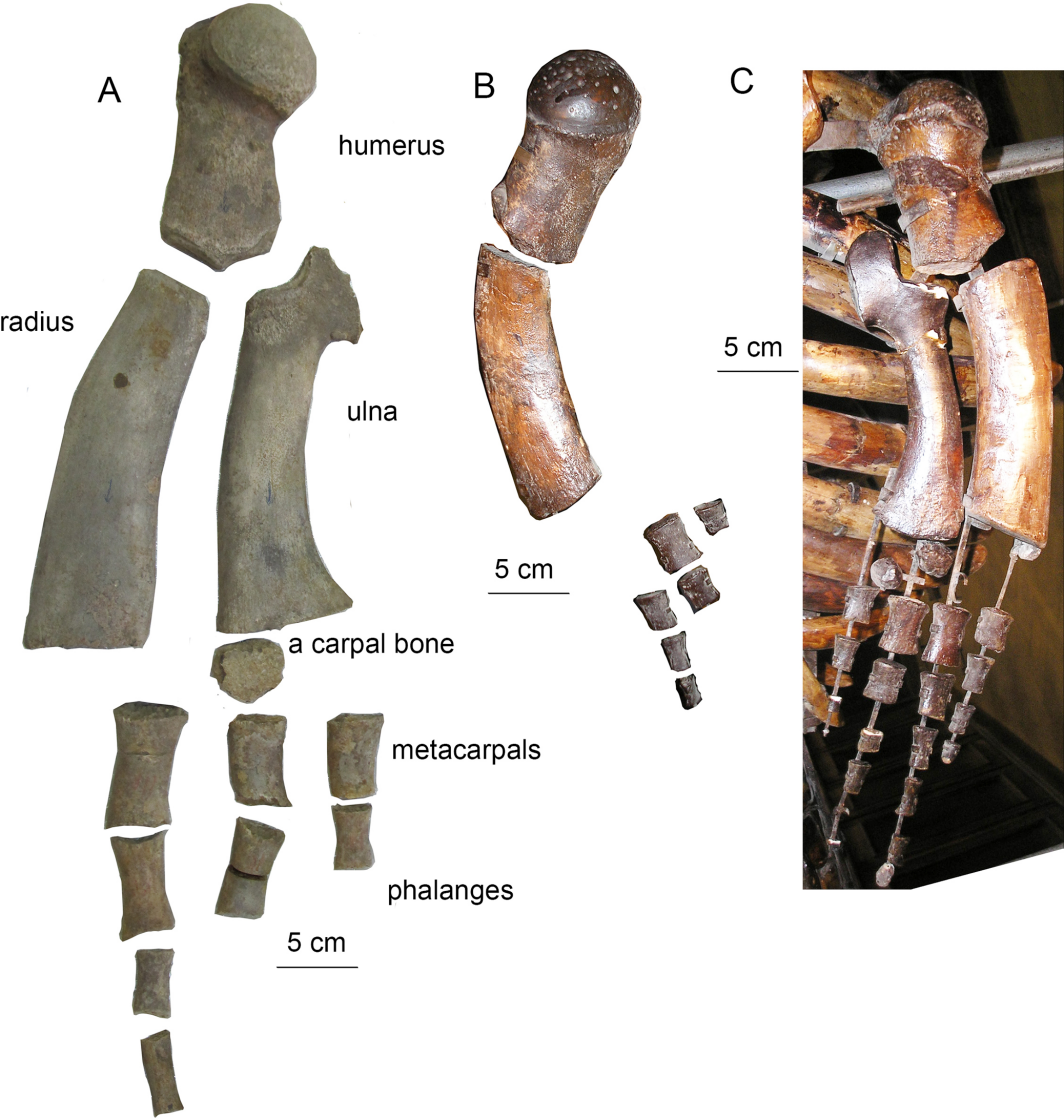
Forelimb bones of *Otradnocetus*. (A) *O. virodovi*, GNM CO-5–12, left forelimb in lateral view; (B) *Otradnocetus* sp., VSEGEI 2401, left forelimb: humerus in posterolateral view; other bones in lateral view; (C) *Otradnocetus* sp., VSEGEI 2401, mounted right forelimb with re-constructed radius and ulna: humerus in posteroventrolateral view; manus bones, lateral view. The scale bars equal five cm.

CETACEA Brisson, 1762
MYSTICETI Flower, 1864
**PLICOGULAE** Geisler et al., 2011
CETOTHERIOIDEA sensu Gol’din & Steeman, 2015
*OTRADNOCETUS VIRODOVI* Mchedlidze, 1984
(Figs. 7–11; Figs. S1–S2; Table S2)

**Holotype.** GNM CO 1–90, a partial skeleton including the incomplete skull, mandible, 43 vertebrae and both forelimbs (humeri, radii, ulnae, a few carpals, metacarpals, and phalanges) with scapulae.

*Emended diagnosis* (see also: Mchedlidze, 1984; Bisconti, Lambert & Bosselaers, 2013). Small four-fingered whales, four to five m long, differing from all the other Plicogulae in the shape of the squamosal: a transversely protruded and dorsoventrally high glenoid part with a high supramastoid crest; a high squamosal prominence; a deep, anteroventromedially facing glenoid fossa; a short and high zygomatic process; a wide postglenoid process with a posterior meatal crest bearing a lateral projection, and a short squamosal cleft. This genus only differs from Cetotheriidae, Tranatocetidae, and *Titanocetus* but is similar to *Parietobalaena* in having a very short ascending process of the maxilla, a short lateral process of the maxilla, an anterior end of nasal located anterior to the rostrum base, and a supraorbital process of the frontal bone directed perpendicular to the anteroposterior axis of the skull. It differs from Cetotheriidae and Balaenopteridae but similar to *Parietobalaena* and Tranatocetidae in presence of a concavity on the lateral side of the squamosal; differs from *Diorocetus*, Tranatocetidae, Balaenopteridae, and Eschrichtiidae but similar to *Parietobalaena* and Cetotheriidae in a narrow and relatively high mandibular condyle (lower than in Cetotheriidae) and a posterior end of the mandible that is oblique to the longitudinal axis of the ramus from the posterior view. Differs from *Parietobalaena* in a supraorbital process of the frontal bone not elongated at the lateral end and a robust medially bent coronoid process of the mandible; further differs from *Parietobalaena* and *Tiucetus* in a paroccipital process extending posterior to the occipital condyle, a distinct glenoid fossa on the medial side of the glenoid part of the squamosal, a short and high zygomatic process, and an anteroposteriorly thick, very robust postglenoid process. Distinct (similar to Cetotheriinae and *Caperea* and different from other Plicogulae) for pachyosteosclerosis of the skeleton, especially developed in vertebrae and ribs.

**Locality and age.** Otradnaya or Urupsky in the northwestern Caucasus (approximate geographical coordinates 44°27′N, 41°33′E). Age between Tarkhanian and Konkian (16.2–12.8 Ma) of the Eastern Paratethys, Middle Miocene. According to the map by Semenova & Uskov (1957), the locality represented by a single lithological unit between Urupsky and Otradnaya is dated as early as Tarkhanian, the stratum corresponding to the latest Burdigalian and early Langhian (16.2–15.3 Ma); however, Mchedlidze (1984) broadly dated it by the Middle Miocene without further evidence. No microfossils are associated with the specimen.

### Description

Due to the fact that the detailed original description written in Russian (Mchedlidze, 1984: 1–64) is largely unknown for a broad readership, a brief re-description is provided here. The skeleton is physically mature, with all the epiphyseal sutures fused to vertebrae and forelimb bones (humerus, radius, and ulna) and mostly obliterated. Cranial sutures of the neurocranium also show an advanced degree of fusion. The **skull** (Fig. 7) is 156 cm long, which is nearly 30% of the body length, the latter being directly estimated at five m from the length of the preserved skeleton (see also: Lambert et al., 2010). Such a proportionally long skull was also previously recorded in *Cetotherium*, as well as in Balaenidae (Gol’din, Startsev & Krakhmalnaya, 2014). After its original description the skull was damaged, and only parts of the rostral portion are now preserved, as can be inferred when comparing the original drawings (Fig. 7). The rostrum is narrow and straight, and the neurocranium is short and wide, with laterally bulged braincase and transversely expanded, particularly wide squamosals. The premaxilla is relatively wide in its rostral portion and not transversely constricted anterior to the nasal. The maxilla (the posterior portion of which has now been lost and can only be seen only on original photographs by Mchedlidze, 1984) has a short lateral process and a short and blunt triangular ascending process. The nasal (now mostly lost) is long, wedge-shaped; its anterior end was anterior to the rostrum base (Fig. 7; Mchedlidze, 1984: fig. 1). The posterior ends of the facial bones do not completely overlap frontals and form a single transverse line at the level of the center of the orbit. The supraorbital process is rectangular from the dorsal view; it is as wide distally as at the base and is directed perpendicular to the longitudinal axis of the skull (Fig. 8A). From the anterior view, it gradually slopes laterally. The parietal is exposed for a small area on the skull vertex (Figs. 8A and 9A). The squamosal (Figs. 8 and 9) is distinct with a very widely transversely protruded glenoid part with postglenoid and zygomatic processes, a high supramastoid crest, a distinct squamosal prominence, and a short squamosal cleft. The deep, anteroventromedially facing glenoid fossa (following the terminology by Marx, Lambert & Uhen, 2016a: 55) is situated on the medial side of the glenoid part of the squamosal; there is also a shallow fossa on its lateral side (like in *Parietobalaena*) and a shallow and wide sternomastoid fossa on the posterior side. The zygomatic process is short, high and robust. The wide postglenoid process bears a posterior meatal crest with a lateral projection (like in Tranatocetidae). The occipital shield has high nuchal crests and a low external occipital crest. The ventral side of the skull is mostly badly damaged; only the occipital area can be seen (Fig. 8B). The compound posterior process of the tympanoperiotic is short, with a neck transversely and dorsoventrally constricted, and its distal end is exposed on the lateral side of the skull in a way similar to *Parietobalaena*. The **mandible** is transversely bowed in dorsal view (Fig. 7), and is heightened at mid-length, as seen in lateral view (see also: Gol’din & Radović, 2018). The posterior portion of the mandible is oblique to the longitudinal axis of the bone: its vertical axis is set at an angle with the axis of the more anterior part of the mandible (Fig. 10). The coronoid process is dorsoventrally low, very robust, and it is medially directed; the mandibular foramen is situated ventrally to it. The mandibular condyle is narrow and high (73.4% of the total posterior height of the mandible and 1.23 times higher than the maximum height of the horizontal ramus) (Table S2), with a deep sub-condylar furrow, and it is intermediate in shape between *Parietobalaena* and Cetotheriidae; the angular process protrudes 10 mm posterior to it. The **vertebral formula**, as preserved, is C_7_Th_12_L_7_Ca_17_, nearly identical to that of *Cetotherium riabinini* (Gol’din, Startsev & Krakhmalnaya, 2014). Mchedlidze (1984) suggested a few lumbar vertebrae were missing, and their total number could be up to 12: however, there is no evidence for such a loss and the lumbar series changes gradual morphologically, although the posteriormost caudal vertebra could be lost. All the vertebrae (including cervicals) are free, except pathological fusion of neural spines of two lumbar vertebrae, L_3_ and L_4_, which are also unusually anteroposteriorly shortened (Fig. S1). All ribs except for the last rib have capitula and tubercles for articulation with the vertebrae. All the vertebrae and ribs show strong pachyosteosclerosis. A **scapula** has both acromion and coracoid processes. A **humerus** has a proportionally large, laterally facing head, a dumbbell-shaped diaphysis, very low tubercles and a reduced deltopectoral crest (Fig. 11A; Fig. S2). There is a large, although incomplete, set of metacarpals and phalanges of digits III–V of the left forelimb (Fig. 11A).

**Figure 12:**
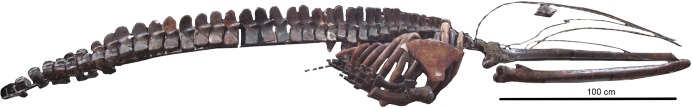
The skeleton of *Otradnocetus* sp., VSEGEI 2401, from the Middle Miocene of the northwestern Caucasus (Russia), right lateral view. The scale bar equals one m. Courtesy of Grigory Prokopov.

**Figure 13:**
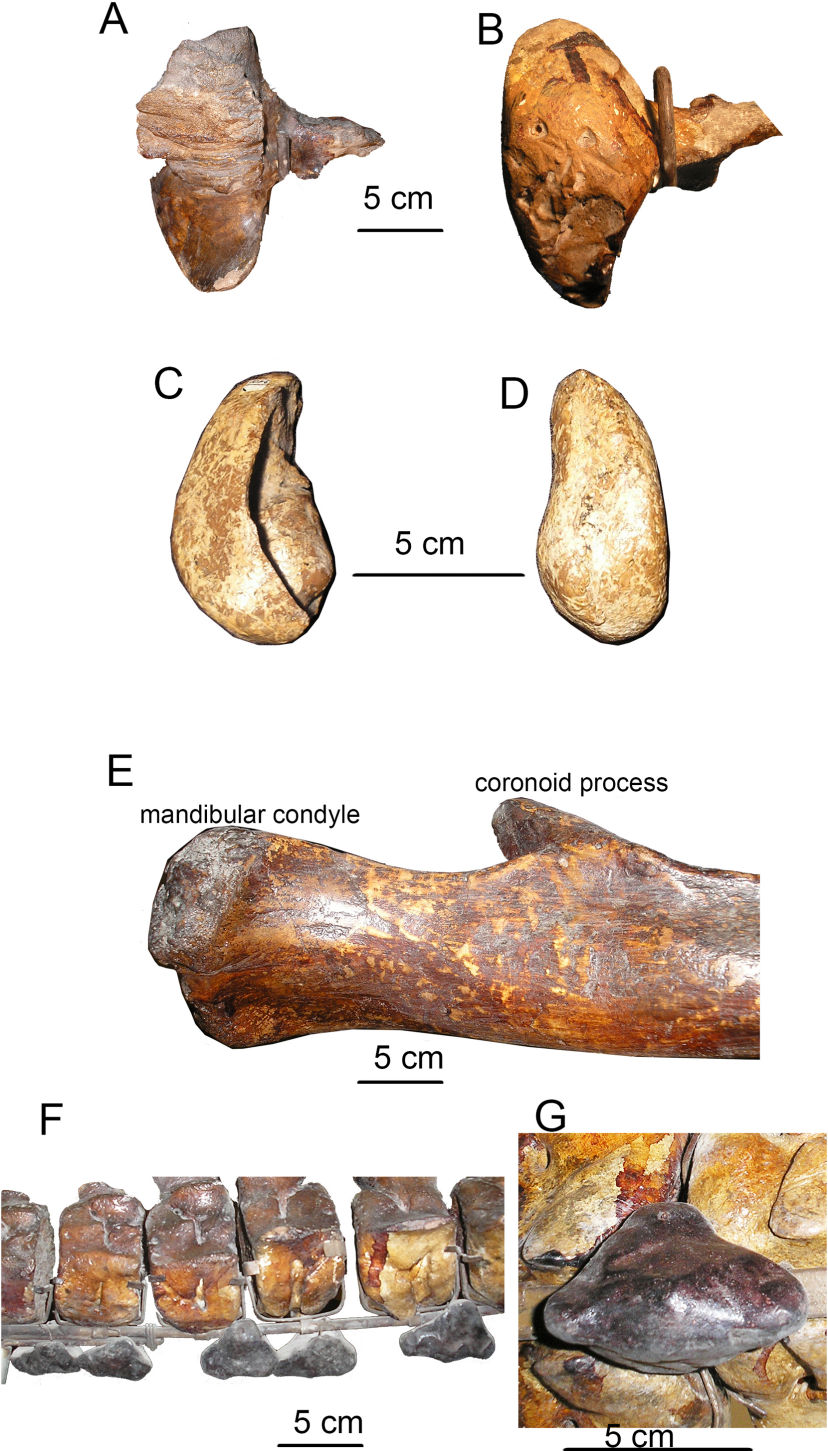
Specific features of skeleton of *Otradnocetus* sp., VSEGEI 2401. (A) Left squamosal, dorsal view; (B) left squamosal, ventral view; (C) left tympanic bulla, ventral view; (D) left tympanic bulla, medial view; (E) posterior portion of the right mandible ramus, lateral view; (F) caudal vertebrae with chevron bones, right lateral view; (G) a chevron bone, ventral view. The scale bars equal five cm.

*OTRADNOCETUS* sp.
(Figs. 11–13; Fig. S3; Table S3)

*Cetotherium* aff. *mayeri* Riabinin, 1934.
?Cetotheriidae indet. Gol’din & Startsev, 2017.

**Specimen.** VSEGEI 2401, a partial skeleton of a juvenile whale including skull fragments, mandibles, cervical, thoracic, lumbar and caudal vertebrae, ribs, scapulae and limb bones (humeri, radii, ulnae, metacarpals, phalanges).

**Locality and age.** Near Kievskoye, in the valley of the Kudako river in the northwestern Caucasus; Chersonian Fm., late Sarmatian s.l. (= Tortonian, between 10 and 7.5 Ma: Radionova et al., 2012; Popov et al., 2013) of the Eastern Paratethys, Late Miocene.

### Description

Detailed original description of the specimen was published in Russian by Riabinin (1934: 1–20) and discussed by Gol’din & Startsev (2017), and here a brief re-description is provided. The specimen is juvenile, with unfused sutures between all the occipital bones that imply an age under 6 months (Walsh & Berta, 2011; Tsai, 2017). All the preserved vertebrae have the centra unfused with the epiphyses; however, the posteriormost caudal vertebrae, which are expected to fuse first (Gol’din & Radović, 2018), are missing. All the forelimb epiphyses are unfused. Meanwhile, all the neural arches are fused with the centra of the vertebrae, an indication for a non-neonate age of at least a few months. Therefore, it is likely that the individual age of the specimen VSEGEI 2401 was only slightly under 6 months. The total body length (measured in situ) is 3.09 m (Riabinin, 1934), suggesting an adult body size between 3.5 and 4 m (Walsh & Berta, 2011; Gol’din & Startsev, 2014; Gol’din & Radović, 2018). The **skull** has been preserved as a few minor fragments, and its general outlines, photographed in situ (Riabinin, 1934), can be re-constructed (Fig. 12). The supraorbital process is rectangular in dorsal view (Fig. S3). The occipital condyles are high and wide, nearly contacting with ventral ends; the foramen magnum is higher than wide. The squamosal, as preserved, is distinct with the wide, ventrolaterally oriented postglenoid process. The **tympanic bulla** (Figs. 13C and 13D; see also Gol’din & Startsev, 2017) is relatively short, 62 mm, anteriorly narrowing from the medial view, with an involucrum thickening posteriorly, and with specific shape as seen from the posterior view, an intermediate state between hexagonal and globular contour, which is a primitive state for Plicogulae (see also: Gol’din & Startsev, 2017). The **mandible** (Figs. 12 and 13E; Fig. S3) is laterally bent in dorsal view and ventrally bent in lateral view. The thick coronoid process, as preserved, is medially oriented; the mandibular foramen is under its base. The condyle is narrow and relatively high, elevated above the ramus, but it is lower than in any cetotheriine or *O. virodovi*: its height is only 55% of the total posterior height of the mandible. The sub-condylar furrow is shallow. The angular process is short and robust, and, unlike in any cetotheriid or *O. virodovi*, it does not go posterior to the condyle. The **vertebral formula**, as preserved, is C_7_Th_10+_L_6+_Ca_17_. Most of vertebrae are damaged, and some of them are re-constructed, obscuring their original anatomy. At least, three thoracic vertebrae are missing (Riabinin, 1937). All the vertebrae show strong pachyosteosclerosis of neural spines and transverse processes. The caudal vertebrae bear robust, pachyostotic chevron bones of irregular shape (Figs. 13F and 13G). The **humerus** (Fig. 11B) has a large globular head and a straight cylindrical diaphysis, with an extremely reduced deltapectoral crest. The radius is nearly straight, only slightly anteroposteriorly curved. A few manus bones have been preserved (Figs. 11B and 11C): on both sides they follow the overall pattern of a tetradactyl manus, the best preserved on the left side with a tentative phalangeal formula: II-3 III-5 IV-4 V-3, which is in the range of extant Plicogulae (Cooper et al., 2007; Cooper, 2009).

#### Comparison with O. virodovi

VSEGEI 2401 differs from *O. virodovi* by a proportionally lower mandibular condyle, 55% of the total posterior height of the mandible (unlike nearly ¾ in *O. virodovi*), a shorter angular process that does not go posterior to the condyle, a proportionally larger humeral head (60% of the total humeral length) and a straight humeral diaphysis, and, presumably, a lesser body length, maximum four m. Due to the fragmentary nature and poor preservation state the specimen VSEGEI 2401 is not formally designated here as a type specimen for a new species. However, it is highly probable that it represents a new species, the validity of which should be proven after finding of more complete, desirably adult specimens.

#### Phylogeny

**Figure 14:**
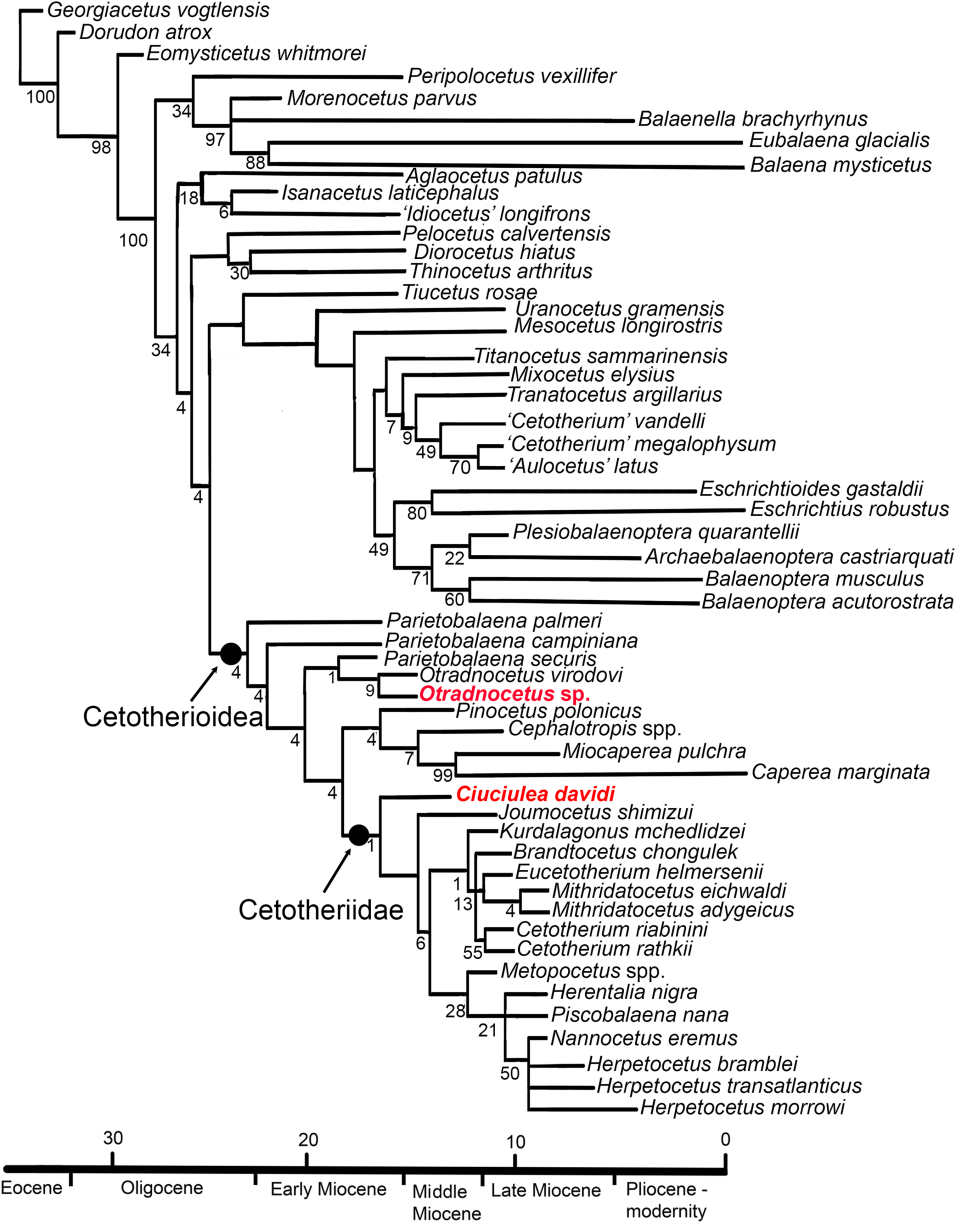
Consensus phylogenetic tree of Neogene and recent Mysticeti, with newly described taxa. Eight trees, best score = 513 steps, CI = 0.32, RI = 0.66. Branch support (GC) values are indicated below the branches.

The phylogeny obtained here (Fig. 14) is similar to the phylogenetic tree published earlier by Gol’din & Startsev (2017). It recovers a monophyletic clade of Plicogulae, with two major lineages: superfamily Cetotherioidea including the extant pygmy right whale *Caperea*, and another lineage containing Balaenopteridae, Eschrichtiidae and Tranatocetidae with more stemward taxa *Uranocetus* and *Tiucetus*. All the other Neogene baleen whales (“cetotheres” s.l.), including *Diorocetus hiatus*, are recovered as basal groups to the crown Plicogulae.

Cetotherioidea include two monophyletic groups, Cetotheriidae *sensu stricto*, and another lineage including *Pinocetus*, *Cephalotropis*, and Neobalaenidae. In addition, Cetotherioidea include basal taxa, most of which are formally described or designated as *Parietobalaena* spp., and *Otradnocetus*. In this tree *Ciuciulea davidi* is the basalmost taxon of Cetotheriidae *s.s*. Within Cetotheriidae, *Ciuciulea davidi* and *J. shimizui* are basal to two major diverse clades including on one side *Cetotherium* and on the other side *Herpetocetus*, *Metopocetus, Herentalia*, and *Piscobalaena*.

Surprisingly, this phylogeny does not find *Tiucetus rosae*, a recently described whale from Peru with cetotheriid affinities (Marx, Lambert & De Muizon, 2017), as a member of Cetotherioidea but the earliest branching member at the base of a major sister clade crowning in Balaenopteridae and Eschrichtiidae. However, the same concerns Tranatocetidae, a group placed within Cetotheriidae in most other studies (Marx, Lambert & De Muizon, 2017). *Tiucetus* and Tranatocetidae, as well as Cetotheriidae, share relatively large compound posterior process of the tympanoperiotic, a posteroventral sulcus close to this process and an enlarged paroccipital concavity. However, these structures can correspond to a similar function, serving for attachment for the hyoid apparatus (Marx, Lambert & De Muizon, 2017), and it is unclear if they are phylogenetically significant. Meanwhile, both *Tiucetus* and Tranatocetidae clearly differ from all the Cetotheriidae in the anatomy of the tympanic bulla (see also: Gol’din & Steeman, 2015). Cetotheriidae, as well as *Parietobalaena* and *Otradnocetus*, share a specific ovoid bulla shape with shallow furrows, a small conical process, a relatively short (but not distinctively short, like in Tranatocetidae) anterior lobe; the bulla anteriorly tapers in medial view, or, rarely it has an oval anterior end; however, it is never dorsoventrally flattened and squared, like in Neobalaenidae or *Tiucetus*. This further indicates the close affinities of *Parietobalaena* and Cetotheriidae (Kellogg, 1924).

Thus, the phylogeny presented here is a hypothesis among a series of recent studies on Neogene mysticetes, which agree little to each other (El Adli, Deméré & Boessenecker, 2014; Bisconti, 2015; Marx, Lambert & De Muizon, 2017, and refs therein): especially controversial is the origin of Neobalaenidae, but a few other points of the tree are highly debatable. Hopefully, the addition of new taxa and combinations of characters from different datasets will progressively lead to convergences between topologies. Therefore, there is need for new works in phylogeny including stem Plicogulae and revision of existing datasets.

## Discussion

### *Ciuciulea* as the basalmost cetotheriid

*Ciuciulea* shares the combination of diagnostic traits of at least three anatomical structures with all the Cetotheriidae (Gol’din, Startsev & Krakhmalnaya, 2014; Gol’din & Startsev, 2017). These include strongly telescoped wedge-shaped facial bones, with the posterior portions of the nasal, premaxilla and maxilla extending toward the tip of the occipital shield (X-shaped vertex); in dorsal view, ascending processes of the maxillae with concave lateral margins, approaching medially at their posterior apices; and an ovoid bulla with shallow lateral and medial furrows and a transversely short sigmoid process. Therefore, it is classified as a member of family Cetotheriidae that is confirmed by this phylogenetic analysis.

However, *Ciuciulea* differs from the rest of Cetotheriidae in the plesiomorphic anatomy of the periotic bone: the pars cochlearis is transversely narrow, and it bears a particularly short posterior cochlear crest. In that feature, *Ciuciulea* is more similar to stem Plicogulae such as *Diorocetus hiatus*, *Amphicetus later*, or the early diverging Cetotherioidea *Parietobalaena* spp. (Steeman, 2007, 2010) and shared by another cetotheriid, *Z. nartorum* from the Bessarabian (the latest Serravallian) of Adygea (Tarasenko, 2014). *Ciuciulea* is also distinct in another plesiomorphic *Diorocetus*-type periotic feature, a pars cochlearis bulging out ventral to fenestra rotunda (see also Steeman, 2007). A skull vertex with a long interparietal region and a sagittal crest is primitive for Mysticeti and, as well as the overall skull size and proportions, is a trait similar to *J. shimizui* and *Parietobalaena palmeri*. A small braincase, which is low and narrow, is a primitive state resembling the earliest toothless mysticetes, Eomysticetidae, and shared by few Middle Miocene taxa, such as *Parietobalaena palmeri*, *Parietobalaena campiniana* and *Imerocetus karaganicus*. Meanwhile, *Ciuciulea* differs from the other Middle Miocene mysticetes in an apomorphic shape of the bulla which is among the best diagnostic traits for baleen whales at the family level (Gol’din & Steeman, 2015): as in the other cetotheriids, the bulla has a short and thick sigmoid process, which is not distinctly swollen at the base, a low conical process, a moderately sized anterior lobe, a shallow sigmoid fissure and a shallow lateral furrow (Gol’din, Startsev & Krakhmalnaya, 2014). Notably, *Ciuciulea* is extremely different in anatomy of the tympanic bulla from *Pinocetus polonicus* (Czyzewska & Ryziewicz, 1976) and *Cephalotropis* spp. (Kellogg, 1940), the former one having been suggested to be closely related to Cetotheriidae (Marx, 2011; Gol’din & Steeman, 2015; this study), and the latter one even as a member of Cetotheriidae (Marx, Lambert & De Muizon, 2017). The ovoid, shallow-furrowed bulla of *Ciuciulea* is typical for cetotheriids, whereas, on the contrary, squared bullae with deep furrows seen in *Pinocetus* and *Cephalotropis* do not fit the diagnosis of this family: Steeman (2007) found this shape more similar to *Aglaocetus*; meanwhile, it is also somewhat similar to *Caperea* (Gol’din, Startsev & Krakhmalnaya, 2014; Gol’din & Steeman, 2015). In the same way, *Ciuciulea* differs in the shape of the tympanic bulla from “*Mesocetus*” *agrami* and “*Cetotherium maicopicum*” from the Miocene of the Paratethys, two poorly known taxa with uncertain affinities (Van Beneden, 1884; Spassky, 1951: fig. 3): both have a large anteriorly squared bulla of a *Aglaocetus*- or *Pinocetus*-type (*sensu* Steeman, 2007). Also, *Ciuciulea* differs from *I. karaganicus*, a small whale having unusual autapomorphic skull anatomy with posteroventral shift of posterior skull region with squamosals and occipital condyles (Mchedlidze, 1964). Finally, *Ciuciulea* differs from *Otradnocetus* (Mchedlidze, 1984) in the distinctive cetotheriid anatomy of its facial bones: the long ascending processes of the maxillae extend posterior on the vertex, by the level of the temporal fossa. Thus, *Ciuciulea davidi* is characterized with a very distinctive set of cranial features, which is easily discriminated from the other Neogene mysticetes.

### *Otradnocetus* as a transitional taxon

*Otradnocetus* has a few distinct autapomorphies in the squamosal and mandibular anatomy. Still *Otradnocetus* is clearly related to *Parietobalaena*, as recognized by Mchedlidze (1984) during its original description. *Otradnocetus* has typical *Parietobalaena*-like anatomy of the dorsal side of the skull, with primitively short ascending processes of maxillae that extend posterior only to the anterior part of orbits, anterior to the temporal fossa; posterior ends of the rostral bones forming a straight transverse line on the skull vertex; and short lateral process of maxilla (Bisconti, Lambert & Bosselaers, 2013). However, most of these similarities are symplesiomorphies. Meanwhile, *Otradnocetus* surprisingly shows some cetotheriid apomorphies, the most important being a relatively high mandibular condyle (55–75% of the total posterior mandible height), which is oblique to the longitudinal axis of the ramus, and a relatively well developed angular process: this anatomy has been recognized as a critical cetotheriid synapomorphy (El Adli, Deméré & Boessenecker, 2014). In addition, it has a cetotheriid-like squamosal with a medially located glenoid fossa, a robust posteriorly directed postglenoid process, and a very short Cetotheriinae-like zygomatic process (Gol’din & Startsev, 2017). The anatomy of its compound posterior process of the tympanoperiotic is also transitional between *Parietobalaena* and Cetotheriidae, and would be sufficient evidence to identify *Otradnocetus* as a member of either of these groups. The anatomy of the tympanic bulla is primitive, non-specific and overall *Parietobalaena*-like (as discussed by Gol’din & Startsev, 2017); however, it lacks any apomorphy distinguishing it from Cetotheriidae. In this regard, some features shared by *Parietobalaena* and Cetotheriidae, like the smooth bulla with shallow furrows and small processes, seem to be paedomorphic traits; and paedomorphosis is not surprising for this lineage rich in dwarf forms (Tsai & Fordyce, 2014; Tsai, 2017). Meanwhile, *Otradnocetus* shows a peramorphic morphology in comparison with other *Parietobalaena*-like forms, with high skull crests and obliterating skull sutures, apparently contradicting the idea of a transition between *Parietobalaena* and Cetotheriidae through paedomorphic forms. *Otradnocetus* also shows highly advanced degree of pachyostosis of postcranial skeleton, seen not only in an adult *O. virodovi* but also in the juvenile VSEGEI 2401. This trait is shared by most Cetotheriinae in the Eastern Paratethys but not *Ciuciulea* or other Cetotheriidae and thus its phylogenetic significance is controversial.

Finally, *Otradnocetus* sp. VSEGEI 2401 from the Late Miocene is one of the latest *Parietobalaena*-like taxa which can only be compared to *Heterocetus affinis* (or *Parietobalaena affinis*) from the Tortonian of Belgium (Steeman, 2010), and thus it is a relict taxon, which survived in an isolated water body, the eastern Paratethys, along with a few Cetotheriinae.

### Origin and biogeography of Cetotheriidae

Cetotheriidae were distributed in the North Atlantic, North and South Pacific, and Paratethys (Fig. S4). Broad ranges are common for *Parietobalaena* and other taxa that are here considered as Cetotherioidea (Fig. S4, based on: Uhen, 2018; Gol’din & Startsev, 2017). However, notably, the earliest and the basalmost member of Cetotheriidae, *Ciuciulea davidi*, comes from the same region and has approximately the same age as *O. virodovi* (Mchedlidze, 1984) and *Pinocetus polonicus* (Czyzewska & Ryziewicz, 1976), which are recognized here as taxa closest to the base of Cetotheriidae. Moreover, the only other cetotheriid that is unambiguously Middle Miocene in age, *Z. nartorum* (Tarasenko, 2014), comes from the same region (see chronology in: Gol’din & Startsev, 2017). It is tempting to suggest the Eastern and Central Paratethys with the Fore-Carpathian Basin of the Middle Miocene as the biogeographical region where Cetotheriidae evolved before their worldwide dispersal and radiation. This hypothesis is supported by the existence of a group of Cetotheriidae that is endemic for the Eastern Paratethys, Cetotheriinae (Gol’din & Startsev, 2017). The find of *Ciuciulea davidi* in the Middle Miocene further supports this view, and places it at the same time and place as the transitional taxon of *O. virodovi*. All this suggests that the Paratethys is an important region for future studies of Cetotheriidae and related taxa.

## Conclusions

The newly found and re-described taxa discussed here are transitional forms toward Cetotheriidae. They uncover the link between the origin of this family and *Parietobalaena* spp., well-known Middle Miocene whales. Interestingly, most of these early diverging taxa, as well as most of the Cetotheriidae, are dwarf forms with paedomorphic anatomy, as well as the most of Cetotheriidae, marking dwarfism and paedomorphosis as main trends in cetotheriid evolution. The reconstruction of phylogeny for Neogene and extant whales supports the idea of *Parietobalaena* as the ancestral morphotype of Cetotheriidae and Neobalaenidae (pygmy right whales) and a divergence time for the two latter clades at the latest in the Middle Miocene. The palaeogeography of the earliest records of Cetotheriidae and their sister groups indirectly indicates their Paratethyan origin.

## Supplemental Information

10.7717/peerj.5800/supp-1 Supplemental Information 1 Fig. S1. Vertebrae of *Otradnocetus virodovi*, GNM CO-49-50. A, atlas, anterior view; B, axis, anterior view; C, eighth thoracic vertebra, anterior view; D, third lumbar vertebra, anterior view; fused fourth and fifth lumbar vertebrae of, left lateral view. The scale bars equal 5 cm.

10.7717/peerj.5800/supp-2 Supplemental Information 2 Fig. S2. The left humerus of *Otradnocetus virodovi*, GNM CO-49-50. A, lateral view; B, medial view; C, posterior view. The scale bar equals 5 cm.

10.7717/peerj.5800/supp-3 Supplemental Information 3 Fig. S3. The supraorbital process of the frontal bone and mandibles of *Otradnocetus* sp., VSEGEI 2401, dorsal view. The scale bar equals 50 cm.

10.7717/peerj.5800/supp-4 Supplemental Information 4 Fig. S4. Geographical and stratigraphic distribution of Cetotherioidea during the Miocene (based on data from: Gol’din & Startsev, 2017; Uhen, 2018). Middle Miocene records are shown as asterisks, and Late Miocene records are shown as crosses.

10.7717/peerj.5800/supp-5 Supplemental Information 5 Table S1. Character-taxon matrix for the phylogenetic analysis.

10.7717/peerj.5800/supp-6 Supplemental Information 6 Table S2. Measurements (mm) of *Otradnocetus virodovi,* GNM CO 1-90. e, estimated value. Measurements in *italics* marked by asterisk * are adopted from Mchedlidze (1984).

10.7717/peerj.5800/supp-7 Supplemental Information 7 Table S3. Measurements (mm) of *Otradnocetus* sp. 1, VSEGEI 2401.

## Institutional Abbreviations

CAS: Californian Academy of Sciences, San Francisco CA, USA
CMT: Central Museum of Taurida, Simferopol, Ukraine
CU: University of Copenhagen, Denmark
GMNH: Gunma Museum of Natural History, Gunma, Japan
GNM: Georgian National Museum, Tbilisi, Georgia
GSM: Georgia Southern Museum, Statesboro GA, USA
MFM: Mizunami Fossil Museum, Gifu, Japan
MGGC: Museo Geologico Giovanni Capellini, University of Bologna, Italy
MGPT: Museo Regionale di Scienze Naturali di Torino, Turin, Italy
MGUH VP: Museum Geologicum Universitatis Hauniensis (Vertebrate Palaeontology), Geological Museum of the University of Copenhagen, Denmark
MLP: Museo de Ciencias Naturales de La Plata, La Plata, Argentina
MPST: Museo Paleontologico “Il Mare Antico,” Salsomaggiore Terme, Italy
MSM: Department of Natural History and Palaeontology, Museum of Southern Jutland, Gram, Denmark
MSU: Zoological Museum of Moscow State University, Russia
MVZ: Museum of Vertebrate Zoology, University of California, Berkeley CA, USA
MZ: Museum of the Earth, Polish Academy of Sciences, Warsaw, Poland
NMG: National Museum of Republic of Georgia, Tbilisi, Georgia
NMB: Natuurmuseum Brabant, Tilburg, Netherlands
NMNH-P: Paleontological Museum of the National Museum of Natural History, Kiev, Ukraine
NMR: Natuurhistorisch Museum Rotterdam, the Netherlands
NMRA: National Museum of the Republic of Adygeya, Maikop, Russia
ONU: Zoological Museum of Odessa National University, Ukraine
PIN: Paleontological Institute, Moscow, Russia
RBINS: Royal Belgian Institute of Natural Sciences, Brussels, Belgium
RGFC: Collection of the Faculty of Mining and Geology, University of Belgrade, Serbia
SBAER: Inventory of Superintendency of Cultural Heritage of Emilia Romagna Region, Piacenza, Italy
SMNS: Staatliches Museum für Naturkunde, Stuttgart, Germany
SPMI: Mining Institute, St. Petersburg, Russia
TNU: Taurida National University, Simferopol, Ukraine
UCMP: Museum of Paleontology, University of California, Berkeley CA, USA
UL: University of Lisbon, Portugal
USNM: National Museum of Natural History, Smithsonian Institution, Washington DC, USA
VSEGEI: Central Scientific Research Geological Survey Museum, Karpinsky Russian Geological Research Institute, St Petersburg, Russia
ZIRM: Museum of Fossil Faunistic Complexes of Moldova, Institute of Zoology of Academy of Sciences of Republic of Moldova, Chisinău, Moldova
ZMA: Zoological Museum, University of Amsterdam, Netherlands.

## Additional Information and Declarations

### Data Availability

The following information was supplied regarding data availability: The specimen ZIRM V 28/1, collected near the village *Ciuciulea* in the Republic of Moldova, is housed by the Museum of Fossil Faunistic Complexes of Moldova, Institute of Zoology of Academy of Sciences of Republic of Moldova, Chisinău, Moldova. The specimen GNM CO 1–90, collected near Otradnaya or Urupsky in the northwestern Caucasus, is housed by the Georgian National Museum, Tbilisi, Georgia. The specimen VSEGEI 2401, collected near the settlement Kievskoye, in the valley of the Kudako river in the northwestern Caucasus, is housed by the Central Scientific Research Geological Survey Museum, Karpinsky Russian Geological Research Institute, St Petersburg, Russia.

### New Species Registration

The following information was supplied regarding the registration of a newly described species: Publication LSID: urn:lsid:zoobank.org:pub:DD10D3E5-EE76-4198-B685-0892C18CB071. Genus, *Ciuciulea* LSID: urn:lsid:zoobank.org:act:3DC65FC9-DA74-4D13-9104-4D4F03742883. Species, *Ciuciulea* davidi LSID: urn:lsid:zoobank.org:act:A4E90DA0-AF0E-48B5-BA81-6E54CB181D33.

## References

[ref-1] Bisconti M (2015). Anatomy of a new cetotheriid genus and species from the Miocene of Herentals, Belgium, and the phylogenetic and palaeobiogeographical relationships of Cetotheriidae s.s. (Mammalia, Cetacea, Mysticeti). Journal of Systematic Palaeontology.

[ref-2] Bisconti M, Lambert O, Bosselaers M (2013). Taxonomic revision of *Isocetus depauwi* (Mammalia, Cetacea, Mysticeti) and the phylogenetic relationships of archaic “cetothere” mysticetes. Palaeontology.

[ref-3] Boessenecker RW (2011). Herpetocetine (Cetacea: Mysticeti) dentaries from the Upper Miocene Santa Margarita Sandstone of Central California. PaleoBios.

[ref-4] Boessenecker RW (2013). Pleistocene survival of an archaic dwarf baleen whale (Mysticeti: Cetotheriidae). Naturwissenschaften.

[ref-5] Bouetel V, De Muizon C (2006). The anatomy and relationships of *Piscobalaena nana* (Cetacea, Mysticeti), a Cetotheriidae s.s. from the early Pliocene of Peru. Geodiversitas.

[ref-6] Brandt JF (1843). De Cetotherio, novo balaenarum familiae genere, in Rossia meridionali anti aliquot annos eff oso. Bulletin de l’Academie imperiale des Sciences de St Petersbourg.

[ref-7] Brandt JF (1872). Bericht uber den bereits vollendeten, druckfertigen theil seiner untersuchungen uber die fossilen and subfossilen Cetaceen Europas. Compte rendu de l’Académie impériale des Sciences de St. Petersbourg.

[ref-8] Brisson MJ (1762). Regnum animale in classes IX. distributum, sive synopsis methodica sistens generalem animalium distributionem in classes IX, & duarum primarum classium, quadrupedum scilicet & cetaceorum, particularem divisionem in ordines, sectiones, genera & species.

[ref-9] Buchholtz EA (2001). Vertebral osteology and swimming style in living and fossil whales (Order: Cetacea). Journal of Zoology.

[ref-10] Buchholtz EA (2011). Vertebral and rib anatomy in *Caperea marginata*: implications for evolutionary patterning of the mammalian vertebral column. Marine Mammal Science.

[ref-11] Cooper LN, Perrin WF, Würsig B, Thewissen JGM (2009). Forelimb anatomy. Encyclopedia of Marine Mammals.

[ref-12] Cooper LN, Berta A, Dawson SD, Reidenberg JS (2007). Evolution of hyperphalangy and digit reduction in the cetacean manus. Anatomical Record: Advances in Integrative Anatomy and Evolutionary Biology.

[ref-13] Czyzewska T, Ryziewicz Z (1976). *Pinocetus polonicus* gen.n., sp.n. (Cetacea) from the Miocene limestones of Pinczow, Poland. Acta Palaeontologica Polonica.

[ref-14] El Adli JJ, Deméré TA, Boessenecker RW (2014). *Herpetocetus morrowi* (Cetacea: Mysticeti), a new species of diminutive baleen whale from the Upper Pliocene (Piacenzian) of California, USA, with observations on the evolution and relationships of the Cetotheriidae. Zoological Journal of the Linnean Society.

[ref-15] Flower WH (1864). Notes on the skeletons of whales in the principal museums of Holland and Belgium, with descriptions of two species apparently new to science. Proceedings of the Zoological Society of London.

[ref-16] Fordyce RE, Marx FG (2013). The pygmy right whale *Caperea marginata*: the last of the cetotheres. Proceedings of the Royal Society B: Biological Sciences.

[ref-17] Geisler JH, McGowen MR, Yang G, Gatesy J (2011). A supermatrix analysis of genomic, morphological, and paleontological data from crown Cetacea. BMC Evolutionary Biology.

[ref-18] Gol’din P, Radović P (2018). A Middle Miocene baleen whale from Bele Vode in Belgrade, Serbia. Rivista Italiana di Paleontologia e Stratigrafia.

[ref-19] Gol’din P, Startsev D (2014). *Brandtocetus*, a new genus of baleen whales (Cetacea, Cetotheriidae) from the late Miocene of Crimea, Ukraine. Journal of Vertebrate Paleontology.

[ref-20] Gol’din P, Startsev D (2017). A systematic review of cetothere baleen whales (Cetacea, Cetotheriidae) from the late Miocene of Crimea and Caucasus, with a new genus. Papers in Palaeontology.

[ref-21] Gol’din P, Startsev D, Krakhmalnaya T (2014). The anatomy of the late Miocene baleen whale *Cetotherium riabinini* from Ukraine systematic palaeontology. Acta Palaeontologica Polonica.

[ref-22] Gol’din P, Steeman ME (2015). From problem taxa to problem solver: a new miocene family, tranatocetidae, brings perspective on baleen whale evolution. PLOS ONE.

[ref-23] Goloboff PA, Farris JS, Nixon KC (2008). TNT, a free program for phylogenetic analysis. Cladistics.

[ref-24] Hír J, Venczel M, Codrea V, Angelone C, Ostende LWVDH, Kirscher U, Prieto J (2016). Badenian and Sarmatian s.str. from the Carpathian area: overview and ongoing research on Hungarian and Romanian small vertebrate evolution. Comptes rendus Palevol.

[ref-25] Kellogg R (1924). Description of a new genus and species of whalebone whale from the Calvert Cliffs, Maryland. Proceedings of the United States National Museum.

[ref-26] Kellogg R (1925). Fossil cetotheres from California. Contributions to Palaeontology from the Carnegie Institution of Washington.

[ref-27] Kellogg R (1940). On the cetotheres figured by Vandelli. Boletime do Labrotorio Mineralogico e Geologico da Universidade de Lisboa.

[ref-28] Kimura T, Hasegawa Y (2010). A new baleen whale (Mysticeti: Cetotheriidae) from the earliest late Miocene of Japan and a reconsideration of the phylogeny of Cetotheres. Journal of Vertebrate Paleontology.

[ref-29] Lambert O, Bianucci G, Post K, De Muizon C, Salas-Gismondi R, Urbina M, Reumer J (2010). The giant bite of a new raptorial sperm whale from the Miocene epoch of Peru. Nature.

[ref-61] Marx FG (2011). The more the merrier? A large cladistic analysis of mysticetes, and comments on the transition from teeth to baleen. Journal of Mammalian Evolution.

[ref-30] Marx FG, Bosselaers MEJ, Louwye S (2016). A new species of *Metopocetus* (Cetacea, Mysticeti, Cetotheriidae) from the late Miocene of the Netherlands. PeerJ.

[ref-60] Marx FG, Fordyce RE (2015). Baleen boom and bust: a synthesis of mysticete phylogeny, diversity and disparity. Royal Society Open Science.

[ref-31] Marx FG, Lambert O, De Muizon C (2017). A new Miocene baleen whale from Peru deciphers the dawn of cetotheriids. Royal Society Open Science.

[ref-32] Marx FG, Lambert O, Uhen MD (2016a). Cetacean Paleobiology.

[ref-33] Mchedlidze GA (1964). Fossil cetaceans of caucasus.

[ref-34] Mchedlidze GA (1984). A Fossil whale from the Miocene deposits of environs of Otradnaya. Metsniereba.

[ref-35] Mead JG, Fordyce RE (2009). The therian skull: a lexicon with emphasis on the odontocetes. Smithsonian Contributions to Zoology.

[ref-36] Murie DJ (1873). On the organization of the caaing whale, *Globiocephalus1 melas*. Transactions of the Zoological Society of London.

[ref-37] Palcu DV, Tulbure M, Bartol M, Kouwenhoven TJ, Krijgsman W (2015). The Badenian–Sarmatian extinction event in the Carpathian foredeep basin of Romania: paleogeographic changes in the Paratethys domain. Global and Planetary Change.

[ref-38] Popov SV, Akhmetiev MA, Golovina LA, Goncharova IA, Radionova EP, Filippova NY, Trubichin VM (2013). Neogene regiostage stratigraphic scale of the south Russia: current state and perspectives. General stratigraphic scale of Russia. Current state and perspectives. Russian conference.

[ref-39] Popov SV, Rögl F, Rozanov AY, Steininger FF, Shcherba IG, Kovac M (2004). Lithological-paleogeographic maps of Paratethys-10 maps late Eocene to pliocene. Courier Forschungsinstitut Senckenberg.

[ref-40] Radionova EP, Golovina LA, Filippova NY, Trubikhin VM, Popov SV, Goncharova IA, Vernigorova YV, Pinchuk TN (2012). Middle-upper Miocene stratigraphy of the Taman Peninsula, Eastern Paratethys. Open Geosciences.

[ref-41] Riabinin AI (1934). New materials on the osteology of *Cetotherium mayeri* Brandt from the Upper Sarmatian of the Northern Caucasus. Trudy Vsesoyuznogo Geologorazvedochnogo Ob’edineniya SSSR.

[ref-42] Riabinin AI (1937). A mounted skeleton of *Cetotherium mayeri* jun. Brandt. Ezhegodnik Vserossiyskogo Paleontologicheskogo Obshchestva.

[ref-43] Rögl VF (1998). Palaeogeographic considerations for Mediterranean and Paratethys seaways (Oligocene to Miocene). Annalen Des Naturhistorischen Museums in Wien.

[ref-44] Sandvik B (2009). Thematic mapping. http://thematicmapping.org.

[ref-45] Semenova EK, Uskov MV (1957). State geological map of the USSR, 1: 1000000. Chart Sheet L-37. Rostov.

[ref-46] Spassky PI (1951). Remains of cetotheres from the Northern Caucasus (Maikop environs). Izvestiya Akademii Nauk Azerbaijanskoy SSR.

[ref-47] Steeman ME (2007). Cladistic analysis and a revised classification of fossil and recent mysticetes. Zoological Journal of the Linnean Society.

[ref-48] Steeman ME (2010). The extinct baleen whale fauna from the Miocene-Pliocene of Belgium and the diagnostic cetacean ear bones. Journal of Systematic Palaeontology.

[ref-49] Steeman ME, Hebsgaard MB, Fordyce RE, Ho SYW, Rabosky DL, Nielsen R, Rahbek C, Glenner H, Sørensen MV, Willerslev E (2009). Radiation of extant cetaceans driven by restructuring of the oceans. Systematic Biology.

[ref-50] Stefanović I (2010). Note on the first fossil remains of a whale from northern Bosnia. Geološki Anali Balkanskoga Poluostrva.

[ref-51] Tarasenko KK (2014). New genera of baleen whales (Cetacea, Mammalia) from the Miocene of the northern Caucasus and Ciscaucasia: 3. *Zygiocetus* gen. nov. (middle Sarmatian, Adygea). Paleontological Journal.

[ref-52] Tsai CH (2017). A Miocene breeding ground of an extinct baleen whale (Cetacea: Mysticeti). PeerJ.

[ref-53] Tsai CH, Fordyce RE (2014). Juvenile morphology in baleen whale phylogeny. Naturwissenschaften.

[ref-54] Uhen MD (2018). Cetacea. https://paleobiodb.org/classic?user=Guest&action=displayPage&page=OSA_9_Cetacea.

[ref-55] Van Beneden PJ (1884). Une baleine fossile de Croatie appartenant au genre mésocète. Mémoires de l Académie Royale des Sciences, des Lettres et des Beaux-Arts de Belgique.

[ref-56] Vernyhorova YuV (2015). Stratigraphic scheme for the Neogene deposits of the Northern Black Sea region and adjacent part of the Ukrainian Shield. Heolohiia ta rudonosnist Ukrainy.

[ref-57] Walsh BM, Berta A (2011). Occipital ossification of balaenopteroid mysticetes. Anatomical Record: Advances in Integrative Anatomy and Evolutionary Biology.

[ref-58] Whitmore FC, Barnes LG (2008). The Herpetocetinae, a new subfamily of extinct baleen whales (Mammalia, Cetacea, Cetotheriidae). Virginia Museum of Natural History.

[ref-59] Wysocka A, Radwański A, Górka M, Bąbel M, Radwańska U, Złotnik M (2016). The Middle Miocene of the Fore-Carpathian basin (Poland, Ukraine and Moldova). Acta Geologica Polonica.

