# Supplementary figures and images for: New Paratethyan dwarf baleen whales mark the origin of cetotheres
Source: PeerJ. 2018 Oct 15;6:e5800. doi: 10.7717/peerj.5800 (PMC6193469; doi:10.7717/peerj.5800)

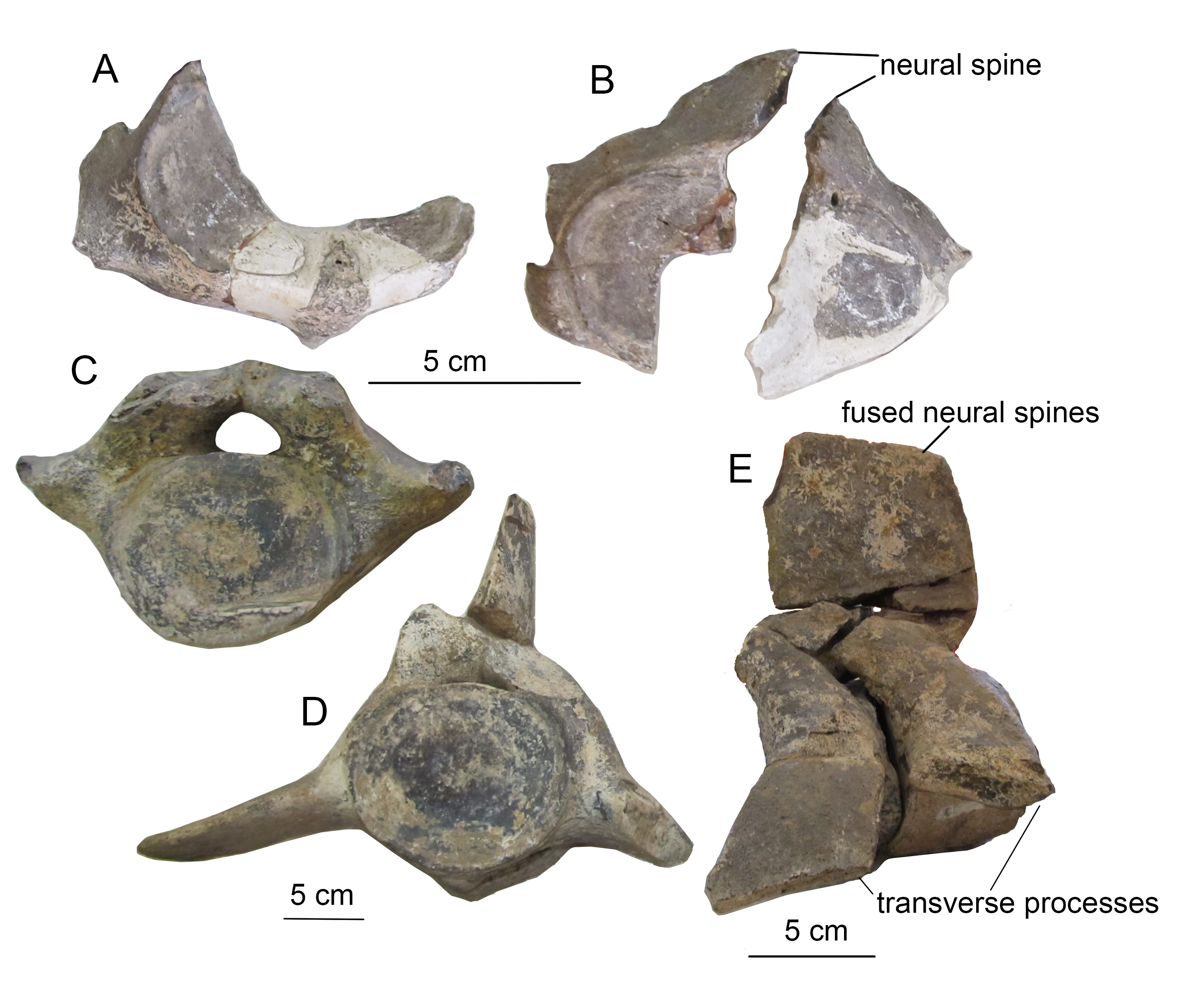

Supplement: Supplemental Information 1 — A, atlas, anterior view; B, axis, anterior view; C, eighth thoracic vertebra, anterior view; D, third lumbar vertebra, anterior view; fused fourth and fifth lumbar vertebrae of, left lateral view. The scale bars equal 5 cm. [file peerj-06-5800-s001.jpg]

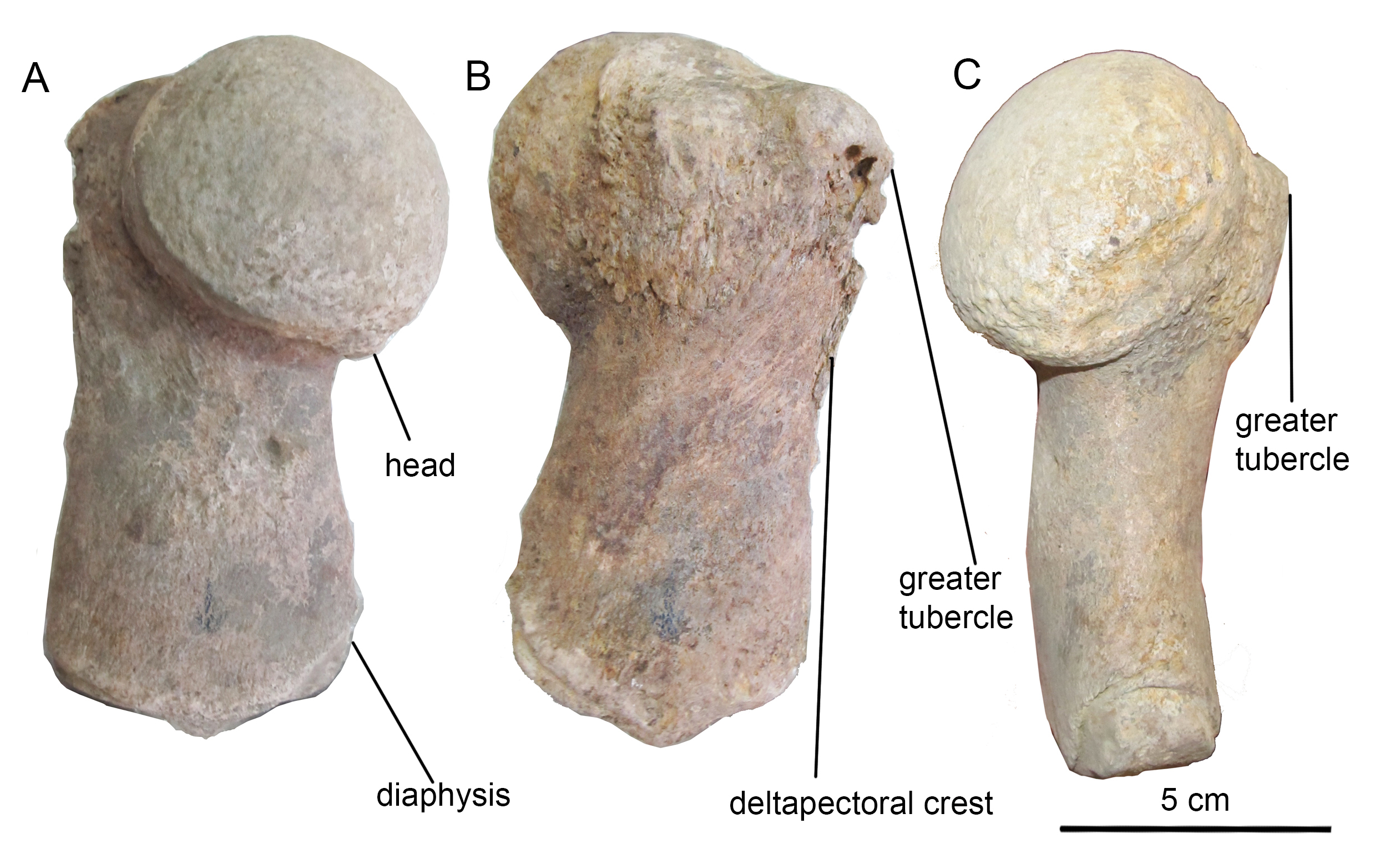

Supplement: Supplemental Information 2 — A, lateral view; B, medial view; C, posterior view. The scale bar equals 5 cm. [file peerj-06-5800-s002.jpg]

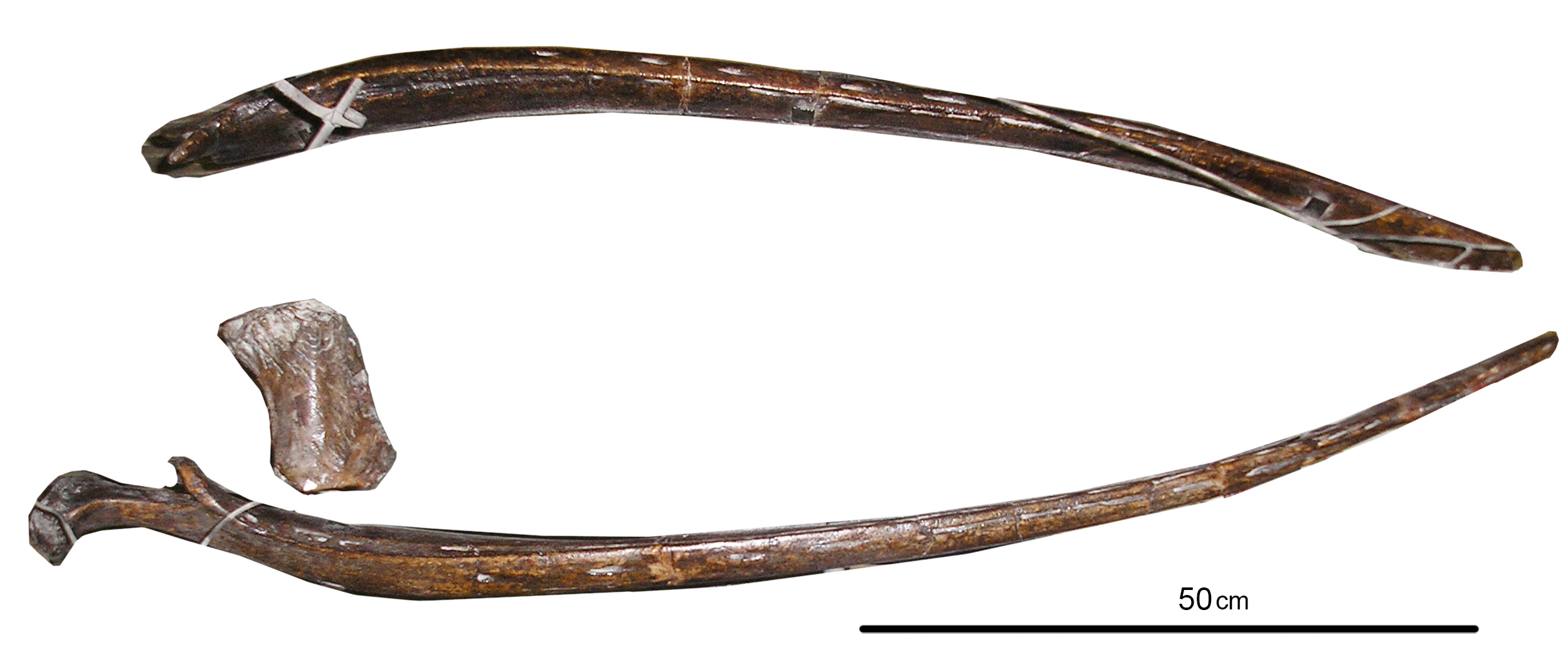

Supplement: Supplemental Information 3 — The scale bar equals 50 cm. [file peerj-06-5800-s003.jpg]

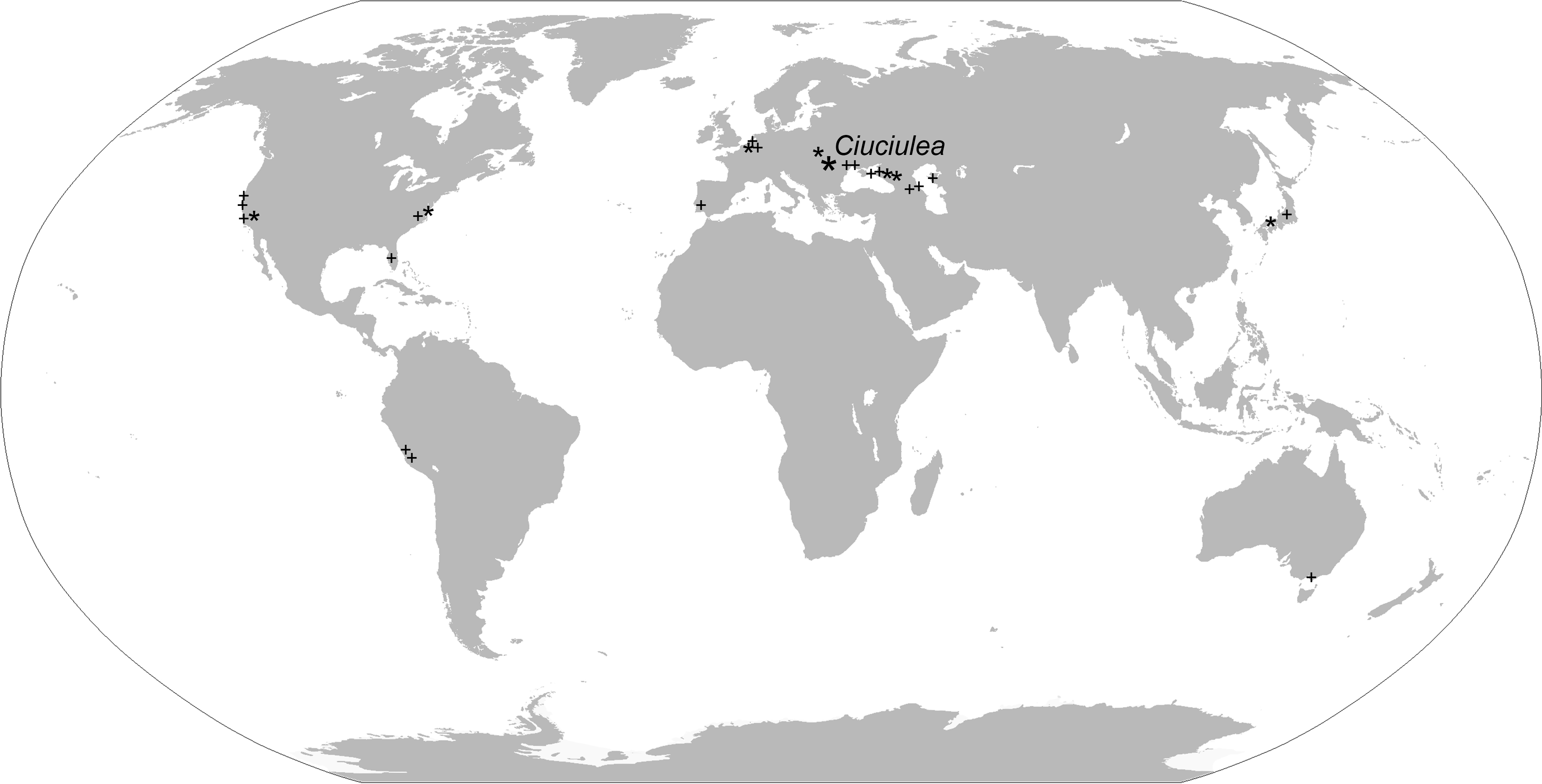

Supplement: Supplemental Information 4 — Middle Miocene records are shown as asterisks, and Late Miocene records are shown as crosses. [file peerj-06-5800-s004.png]
