# Supplementary material for: New Paratethyan dwarf baleen whales mark the origin of cetotheres
Source: PeerJ. 2018 Oct 15;6:e5800. doi: 10.7717/peerj.5800 (PMC6193469; doi:10.7717/peerj.5800)
Supplement: Supplemental Information 5 [file peerj-06-5800-s005.docx]

Table S1. Character-taxon matrix for the phylogenetic analysis.

Georgiacetus_vogtlensis 00000?0000 0000000000 0000000000 0 000000000 000 000000 0 0000000000 000 00 000000 000000 00000000000 0000 ??????????

Dorudon_atrox 00000?0000 0000000000 0000000000 0 000000000 000 100000 0 0000000000 000 00 000000 000000 00000000000 0120 0000000000

Eomysticetus_whitmorei 1110101000 0000000100 0000000000 0 000000000 000 100000 0 0000000000 000 00 000000 0?0000 00000000000 01?0 ???000100?

Aglaocetus_patulus 1110311100 1011000000 1111110000 0 011002111 011 1011?1 0 0101101010 100 10 123110 000100 ??????????? 1??? ??????????

Archaebalaenoptera_castriarquati 1110201110 2031120001 2111111?00 0 ?20002020 01? ???1?? ? ?????????? ??? ?? ?????? ?????? 11012101001 ???? ??????????

Aulocetus_latus 1110??1221 3121221101 11111001?0 1 121021110 101 102110 ? ????21???? ??? ?? 112010 011012 ??????????? ???? ??????????

Balaena_mysticetus 1112101100 1012000200 1112110030 0 022012001 121 100101 2 0200302000 100 00 101010 000011 03312220100 0011 0001010110

Balaenella_brachyrhynus 11111011?0 1?12002200 1112100030 0 020012001 011 100101 2 02001020?? 1?? 00 0110?? 0?00?1 ??????????? ???? ??????????

Balaenoptera_acutorostrata 1110101120 2131110001 2112111120 0 100002010 011 311111 1 0201301100 200 21 110000 100002 11012101001 1010 0102111101

Balaenoptera_musculus 1110211120 2131110001 2112111120 0 100002010 011 311111 1 0201301100 200 21 110000 100002 11012101001 1110 0002111101

Brandtocetus_chongulek ??????11?1 212?111110 1111102?1? 0 020021120 002 1?1?00 0 0101241001 110 00 100000 001002 12111211011 0010 0112102101

Caperea_marginata 1111101000 0111022011 2112113131 1 110?23010 002 200100 0 0100221000 100 00 123110 000110 14112220?01 1100 0102102101

Cephalotropis_sp 11?0??1110 20211200?? 111001?10? ? ?????1110 001 101100 0 01??221??? 1?? ?? 113110 000112 ??????????? ???? ??????????

Cetotherium_megalophysum 11????1221 312122???? 11110001?0 ? 121021110 101 1????0 0 01012111?0 100 00 112010 011012 ??????????? ???? ??????????

Cetotherium_rathkii 1110111121 2121111110 1111101011 0 020121110 002 101110 0 01012310?1 110 00 110000 000002 ??????????? ???? ??????????

Cetotherium_riabinini 1110111111 2121111110 1111101011 0 021121110 002 111110 0 ????23???? ??? ?? 110000 000002 12111211111 0000 0102102101

Cetotherium_vandelli 1110??1211 3121220111 1111100??0 0 ?10021111 101 212110 ? ????21???? ??? ?? ?????? ?????? ??????????? ???? ??????????

Diorocetus_hiatus 1110311101 1111100110 1110000000 0 010001110 001 101100 1 1011101000 101 01 110000 000002 01001200111 101? 000?????0?

Eschrichtius_robustus 1111211110 2121110000 2111101120 0 020001011 002 311110 0 0003101101 100 00 110000 010000 13212231101 0010 0002101101

Eschrichtioides_gastaldii 11111?1111 2021110000 2111110?20 0 020001011 001 311110 ? ????1????? ??? ?? ?????? ?????? 13212131101 0??? ???211110?

Eubalaena_glacialis 1112101100 1012000200 1112110030 0 022012001 021 100101 2 0200302010 100 00 112010 000011 03312220100 0110 1001010110

Eucetotherium_helmersenii 1110??1210 2121111110 ?1?2100010 1 022121?1? 002 ?????0 0 0?0?221??? ??? ?? ?????? ?????? ???1??1???? ???? ??????????

Herentalia_nigra ??????01?2 212?2????0 ?111100??? 0 ?20021010 001 1??1?0 0 01??221??? ??? ?? ?????? ?????? ??????????? ???? ??????????

Herpetocetus_bramblei 1110110102 112?221110 1111103001 1 012121110 001 101100 0 0101221010 110 00 100000 001002 12112211111 ???? ??????????

Herpetocetus_transatlanticus ??????01?2 112?22???0 1111?03001 1 0121211?0 001 1????0 0 01012210?? 110 00 100000 001002 ??????????? ???? ??????????

Herpetocetus_morrowi 11101?0102 0121221010 1111103001 1 012121120 001 101100 0 01???21010 110 ?? 100000 001002 12112211111 ???? ????1??1??

Idiocetus_longifrons 111???1110 1?1?00???? ?110001010 0 000002120 011 1????1 2 0101100000 100 ?0 11311? 001010 ??????????? ???? ??????????

Isanacetus_laticephalus 1111211100 1011000100 1110101000 0 010001110 011 101101 1 0101101001 100 10 113110 000010 ??????????? ???? ??????????

Joumocetus_shimizui 1110?11111 1121111110 11110010?0 0 0100?1110 011 1????0 0 010?21???0 1?? ?? 11000? 0010?2 ??????????? ???? ??????????

Kurdalagonus_mchedlidzei ?110??11?1 2121121110 1111101110 0 020021120 001 1??1?0 ? 0101251001 110 00 ?????? ?????? ??????????? ???? 010???????

Tranatocetus_argillarius 111???12?1 3?2??2???0 1110002100 0 010001121 102 101110 0 110?11?1?? 1?0 00 112010 011012 010012110?? 01?? 021?0011??

Mesocetus_longirostris 111???11?1 ??2?11???0 ??11010100 0 11000?120 ?01 2????0 ? ????140??? ??? ?? ?????? ?????? 0??0??0?0?? ???? ??????????

Metopocetus_sp ??????01?2 212?12???? 11111010?1 0 120021?10 002 1??1?0 0 0103241010 110 00 100000 001002 ??????????? ???? ??????????

Miocaperea_pulchra 1111101000 011?0?2020 2112113131 1 110?23001 001 2?0100 0 010?2210?? 1?? 00 ?????? ?????? ??????????? ???? ??????????

Mixocetus_elysius 1110211220 2011121100 1111100?00 0 110001001 102 ?????? ? ?????????? ??? ?? 11201? 0?10?? 11012?0100? ???? ??????????

Morenocetus_parvus ??????11?0 1?12002200 1112100030 0 020012001 021 100101 2 02??30???? 2?? 00 ?????? ?????? ??????????? ???? ??????????

Nannocetus_eremus ?????????2 ??2??????0 1111?03021 1 012121?2? 001 2??100 0 0101221?10 110 00 100000 001012 ??????????? ???? ??????????

Otradnocetus_virodovi 1110111100 1011000100 1110001110 0 111022120 012 1??1?0 ? ????1????? ??? ?? ?????? ?????? 12111211111 ?01? 0??2112101

Parietobalaena_campiniana 11101111?0 1?11000011 111???1110 0 01000??2? ?01 111100 1 0101111000 101 00 11000? 0000?0 12001101111 1??? ??????????

Parietobalaena_palmeri 1110311100 1111000100 1110000100 0 110001110 011 111100 0 0102111000 100 00 120001 001010 02001101111 001? ??????????

Parietobalaena_securis 11101111?? ??11000110 1110??1?00 0 12100???? ?02 1?1100 0 0101241000 100 00 ?????? ?????? ??????????? ???? ??????????

Pelocetus_calvertensis 11103111?0 1?11001110 1110100000 0 010001110 021 102111 1 020?1011?0 1?? 00 110001 000010 01011101001 000? 20021021?0

Peripolocetus_vexifiller ?????????? ?????????? ?112101000 0 010002021 021 1???00 2 00001100?? 100 00 110000 000012 ??????????? ???? ??????????

Pinocetus_polonicus 111??11110 2011111110 1110000?0? 0 ?????1110 ??? ?021?? ? ?????????? ??? ?? 123010 000002 1?11??01??? 001? 01121021??

Piscobalaena_nana 1110210122 2121221010 1111100001 0 020121020 002 111100 0 0101221010 110 00 100000 011012 12112211111 001? 0202102101

Plesiobalaenoptera_quarantellii 11102111?0 ??311200?1 21???????? ? ????????? ??? ?????? 0 0001301110 200 21 110000 101002 11012101001 1110 ??????????

Thinocetus_arthritus ?????????? ?????????? ?????????0 0 01000???0 ?01 1????0 1 1011101000 101 10 110000 100000 01001201011 0010 0112102101

Titanocetus_sammarinensis 1110211100 2021211101 1111002?01 0 110001001 002 10?1?1 ? ????11???? ??? ?? ???00? ?????0 11012101001 1??? ??????????

Uranocetus_gramensis 1110211102 0121102100 1110101?00 0 110002120 022 102111 ? 10?11111?0 101 ?1 100000 100002 ?1001101101 1??0 000211210?

Mithridatocetus_eichwaldi ??????11?1 212?11???0 ?1?110?1?? ? ?????1110 002 1?1?00 0 0101241101 110 00 100000 001002 ??????????? ???? ??????????

Mithridatocetus_adygeicus ??????11?1 212??2???0 ?1?01001?? 0 022021110 002 1?1?00 0 0101241101 110 00 10000? 001002 ??????????? ???? ??????????

Ciuciulea_davidi 11????1110 212?12???? 11?000?0?? ? ?????1110 0?1 11?1?0 0 11??2??1?? 1?? ?? 100000 001012 ??????????? ?0?? 0???1021??

Tiucetus_rosae 1110??1100 111?00???0 1110101100 0 111001120 101 1??110 0 000?22?1?? 1?? 00 120000 000002 ??????????? ???? ??????????

Otradnocetus_sp_1 ?????????? ??????0100 1????????? 0 1110????? ??? 1????? ? ?????????? ??? ?? 10?0?? ??00?0 12111211101 ?01? ???211210?
