# Supplementary material for: New Paratethyan dwarf baleen whales mark the origin of cetotheres
Source: PeerJ. 2018 Oct 15;6:e5800. doi: 10.7717/peerj.5800 (PMC6193469; doi:10.7717/peerj.5800)
Supplement: Supplemental Information 6 — e, estimated value. Measurements in italics marked by asterisk * are adopted from Mchedlidze (1984). [file peerj-06-5800-s006.docx]

Table S2

Measurements (mm) of *Otradnocetus virodovi,* GNM CO 1-90.

| Measurement | Distance, mm |
| --- | --- |
| Condylobasal length | *1560** |
| Zygomatic width | 570 |
| Rostrum length | 1250 |
| Length of neurocranium (measured from the transverse line joining the antorbital notches to the occipital condyle) | 308 |
| Skull width between antorbital notches | 300 |
| Length of nasal | *109** |
| Distal width of nasal | *15** |
| Greatest width of nares |  |
| Distance between posterior margin of the nasal and anteriormost point of occipital shield | 70 |
| Anteroposterior length of parietal exposure on skull vertex | 12 |
| Skull width across lateral processes of the maxillae | 410 |
| Skull width across preorbital processes of the frontals | 402 |
| Orbit length | 105 |
| Minimum intertemporal width | 149 |
| Greatest length/width of temporal fossa | 91/165 |
| Distance between tip of zygomatic process and tip of postglenoid process | 194 |
| Point-to-point distance between the dorsal margin of foramen magnum and anteriormost point of occipital shield | 159 |
| Maximum distance between outer margins of nuchal crests | 297 |
| Distance between posteriormost points of the paroccipital processes | 320 |
| Width of foramen magnum | 44 |
| Height of foramen magnum | 66 |
| Bicondylar width | 106 |
| Condylar height | 73 |
| Distance between lateral margins of the basioccipital crests | 96 |
| Greatest height of the neurocranium | 167 |
| Postglenoid width | 470 |
| Length of the compound process of the petrotympanic | 100 |
| Length of mandible | 1480 |
| Posterior height of mandible at the condyle and angular process | 143 |
| Height of mandibular condyle | 105 |
| Distance between the mandibular condyle and tip of the coronoid process | 175 |
| Length of the coronoid process | 135 |
| Height of the coronoid process | 113 |
| Length of the angular process | 35 |
| Distance between the mandibular condyle and the mandibular foramen | 222 |
| Height of the ramus, 50 cm from the anterior end | 79 |
| Height of the ramus, 50 cm from the anterior end | 85 |
| Length of humerus (left/right) | 198/203 |
| Proximal width of humerus (left/right) | 104/103 |
| Distal width of humerus (left/right) | 93/88 |
| Length of radius (left/right) | 288/289 |
| Proximal width of radius (left/right) | 65/63 |
| Distal width of radius (left/right) | 89/91 |
| Length of ulna (anterior margin) (left/right) | 264/270 |
| Length of ulna (posterior margin) (left/right) | 294/290 |
| Length of olecranon (left/right) | 83/76 |
| Length of metacarpale II (left/right) | 65/- |
| Length of metacarpale III (left/right) | 87/89 |
| Length of metacarpale IV (left/right) | 65/70 |
| Length of metacarpale V (left/right) | 53/52 |
| Centrum length/width/height, vertebra C1 | 35/142/- |
| Centrum length/width/height, vertebra C2 | 26/154/- |
| Centrum length/width/height, vertebra C3 | 16/70/- |
| Centrum length/width/height, vertebra C4 | 18/66/66 |
| Centrum length/width/height, vertebra C5 | 21/82/63 |
| Centrum length/width/height, vertebra C6 | 19/73/66 |
| Centrum length/width/height, vertebra C7 | 23/79/63 |
| Centrum length/width/height, vertebra T1 | 23/69/63 |
| Centrum length/width/height, vertebra T2 | 31/-/57 |
| Centrum length/width/height, vertebra T3 | 39/66/64 |
| Centrum length/width/height, vertebra T4 | 39/74/66 |
| Centrum length/width/height, vertebra T5 | 39/76/64 |
| Centrum length/width/height, vertebra T6 | 45/79/62 |
| Centrum length/width/height, vertebra T7 | 48/72/68 |
| Centrum length/width/height, vertebra T8 | 51/77/66 |
| Centrum length/width/height, vertebra T9 | 51/81/68 |
| Centrum length/width/height, vertebra T10 | 53/86/68 |
| Centrum length/width/height, vertebra T11 | 58/81/69 |
| Centrum length/width/height, vertebra T12 | 58/81/69 |
| Centrum length/width/height, vertebra L1 | 61/78/69 |
| Centrum length/width/height, vertebra L2 | 64/79/74 |
| Centrum length/width/height, vertebra L3 | 68/83/76 |
| Centrum length/width/height, vertebra L4 | 65/82/77 |
| Centrum length/width/height, vertebra L5 | 62/86/79 |
| Centrum length/width/height, vertebra L6 | 65/98/80 |
| Centrum length/width/height, vertebra L7 | 72/102/82 |
| Centrum length/width/height, vertebra L8 | 72/102/82 |
| Centrum length/width/height, vertebra L9 | 82/103/82 |
| Centrum length/width/height, vertebra Ca1 | 87/102/80 |
| Centrum length/width/height, vertebra Ca2 | 75/98/75 |
| Centrum length/width/height, vertebra Ca3 | 80/102/78 |
| Centrum length/width/height, vertebra Ca4 | 79/98/90 |
| Centrum length/width/height, vertebra Ca5 | 78/94/90 |
| Centrum length/width/height, vertebra Ca6 | 75/94/89 |
| Centrum length/width/height, vertebra Ca7 | 78/91/86 |
| Centrum length/width/height, vertebra Ca8 | 78/83/80 |
| Centrum length/width/height, vertebra Ca9 | 72/73/78 |
| Centrum length/width/height, vertebra Ca10 | 61/63/70 |
| Centrum length/width/height, vertebra Ca11 | 60/56/55 |
| Centrum length/width/height, vertebra Ca12 | 40/55/52 |
| Centrum length/width/height, vertebra Ca13 | 33/46/48 |
| Centrum length/width/height, vertebra Ca14 | 27/38/42 |
| Centrum length/width/height, vertebra Ca15 | 19/33/35 |
| Centrum length/width/height, vertebra Ca16 | 18/23/30 |
| Centrum length/width/height, vertebra Ca17 | 12/18/25 |

e, estimated value. Measurements in *italics* marked by asterisk * are adopted from Mchedlidze (1984).
