# Supplementary material for: New Paratethyan dwarf baleen whales mark the origin of cetotheres
Source: PeerJ. 2018 Oct 15;6:e5800. doi: 10.7717/peerj.5800 (PMC6193469; doi:10.7717/peerj.5800)
Supplement: Supplemental Information 7 [file peerj-06-5800-s007.docx]

Table S3

Measurements (mm) of *Otradnocetus* sp. 1*,* VSEGEI 2401.

| Measurement | Distance, mm |
| --- | --- |
| Length of orbit | 73 |
| Condylar (bicondylar) width | 97 |
| Length of mandible | 1225 |
| Posterior height of mandible | 96 |
| Height of mandibular condyle | 52 |
| Length of tympanic bulla | 62 |
| Length of humerus (right) | 131 |
| Proximal width of humerus | 81 |
| Distal width of humerus | 60 |
| Length of radius (right) | 181 |
| Proximal width of radius | 75 |
| Distal width of radius | 62 |
| Length of metacarpale II | 27 |
| Length of metacarpale III | 24 |
| Length of metacarpale IV | 19 |
| Length of metacarpale V | 15 |
| Length of phalanx II-1 | 36 |
| Length of phalanx III-1 | 28 |
| Length of phalanx IV-1 | 26 |
| Length of phalanx V-1 | 19 |
| Centrum length/width/height, vertebra T3 | 22/67/55 |
| Centrum length/width/height, vertebra T4 | 26/70/57 |
| Centrum length/width/height, vertebra T5 | 26/72/57 |
| Centrum length/width/height, vertebra T6 | 26/77/57 |
| Cenrum length/width/height, vertebra TA | 37/-/- |
| Centrum length/width/height, vertebra TB | 37/-/- |
| Centrum length/width/height, vertebra TC | 37/-/- |
| Centrum length/width/height, vertebra TD | 37/-/- |
| Centrum length/width/height, vertebra Ca1 | 63/75/65 |
| Centrum length/width/height, vertebra Ca2 | 61/71/65 |
| Centrum length/width/height, vertebra Ca3 | 58/71/65 |
| Centrum length/width/height, vertebra Ca4 | 56/68/65 |
| Centrum length/width/height, vertebra Ca5 | 54/65/65 |
| Centrum length/width/height, vertebra Ca6 | 52/65/65 |
| Centrum length/width/height, vertebra Ca7 | 48/64/65 |
| Centrum length/width/height, vertebra Ca8 | 46/55/63 |
| Centrum length/width/height, vertebra Ca9 | 41/50/50 |
| Length/width/height of the first chevron bone | 61/41/38 |
